# Supplementary material for: Potential gains in life expectancy by attaining daily ambient fine particulate matter pollution standards in mainland China: A modeling study based on nationwide data
Source: PLoS Med. 2020 Jan 17;17(1):e1003027. doi: 10.1371/journal.pmed.1003027 (PMC6968855; doi:10.1371/journal.pmed.1003027)
Supplement: S1 STROBE Checklist — STROBE, Strengthening the Reporting of Observational Studies in Epidemiology. (DOCX) [file pmed.1003027.s001.docx]

**S1 STROBE Checklist. The checklist of STROBE guidelines.**

|  | Item No. | Recommendation | Relevant content in this paper |
| --- | --- | --- | --- |
| **Title and abstract** | 1 | (*a*) Indicate the study’s design with a commonly used term in the title or the abstract | In Title: ‘a modelling study based on nationwide data’. |
|  |  | (*b*) Provide in the abstract an informative and balanced summary of what was done and what was found | In the Methods and Findings section of Abstract. |
| Introduction | | |  |
| Background/rationale | 2 | Explain the scientific background and rationale for the investigation being reported | In paragraph 1-4, Introduction. |
| Objectives | 3 | State specific objectives, including any prespecified hypotheses | In paragraph 5, Introduction. |
| Methods | | |  |
| Study design | 4 | Present key elements of study design early in the paper | In paragraph 1, Methods: “This is a nationwide modelling study based on a time series analysis.”. Details of study design are presented in the Statistical analyses section of Methods and the S1 Text. |
| Setting | 5 | Describe the setting, locations, and relevant dates, including periods of recruitment, exposure, follow-up, and data collection | In paragraph 1, Methods: “The daily time-series mortality data on non-accidental causes in 72 Chinese cities (S1 Table) for the period of January 18, 2013 through December 31, 2016 were selected for this study, and a total of 1,226,849 non-accidental deaths were recorded. The data were extracted from the Death Surveillance Points System (DSP) of China.”. |
| Participants | 6 | (*a*) *Cohort study*—Give the eligibility criteria, and the sources and methods of selection of participants. Describe methods of follow-up  *Case-control study*—Give the eligibility criteria, and the sources and methods of case ascertainment and control selection. Give the rationale for the choice of cases and controls  *Cross-sectional study*—Give the eligibility criteria, and the sources and methods of selection of participants | In paragraph 1, Methods: “The data from the DSP have been widely used in the assessment of health risk factors or disease burden and policy formulation. These cities were selected based on the following process: (1) they were randomly selected using a multistage stratification approach that took the sociodemographic characteristics of the Chinese population into consideration; (2) the daily morality counts in these cities were temporally stable without large fluctuations, and no change in the administrative divisions occurred during the study period; and (3) their air pollution and meteorological records were accessible during the study period.”. |
|  |  | (*b*) *Cohort study*—For matched studies, give matching criteria and number of exposed and unexposed  *Case-control study*—For matched studies, give matching criteria and the number of controls per case | NA  NA |
| Variables | 7 | Clearly define all outcomes, exposures, predictors, potential confounders, and effect modifiers. Give diagnostic criteria, if applicable | In the Methods section: **Outcomes** are the city-specific daily counts of mortality, which we used the life expectancy in the corresponding years to calculate the YLL for each death by matching age and sex to the Chinese national life table, which was obtained from the WHO’s website, and then summed the YLLs for all deaths on each day of the study period to compute the daily YLLs of each city. **Exposures**, e.g. concentrations of air pollutants: “The 24-hour mean concentrations of ambient PM_2.5_, SO_2_, NO_2_ and the maximum 8-hour mean levels for O_3_ were averaged from all available monitoring data within each city.”. **Covariates** are presented S2 Table: The list of model parameters in this study. |
| Data sources/ measurement | 8* | For each variable of interest, give sources of data and details of methods of assessment (measurement). Describe comparability of assessment methods if there is more than one group | In the Methods section. **Mortality variables**: “The data were extracted from the Death Surveillance Points System (DSP) of China, which is operated by the National Center for Chronic and Noncommunicable Disease Control and Prevention, Chinese Center for Disease Control and Prevention”; **Air pollution and meteorological variables**: “Daily concentrations for ambient PM_2.5_ and other air pollutants [including sulfur dioxide (SO_2_), nitrogen dioxide (NO_2_), and ozone (O_3_)] were obtained from the China’s National Real-time Publishing Platform for Daily Air Quality (<http://106.37.208.233:20035>), which delivered the real-time concentrations of ambient air pollutants that were measured by state controlled air monitoring stations”; |
| Bias | 9 | Describe any efforts to address potential sources of bias | In the Sensitivity analyses section, Methods. |
| Study size | 10 | Explain how the study size was arrived at | In paragraph 1, Methods: “The mortality data on non-accidental causes in 72 Chinese cities (S1 Table) for the period of January 18, 2013 through December 31, 2016 were selected for this study, and a total of 1,226,849 non-accidental deaths were recorded.”. |
| Quantitative variables | 11 | Explain how quantitative variables were handled in the analyses. If applicable, describe which groupings were chosen and why | In Statistical analysis section, Methods. All the quantitative variables were treated as continuous variables. |
| Statistical methods | 12 | (*a*) Describe all statistical methods, including those used to control for confounding | In Statistical analyses section, Methods. |
|  |  | (*b*) Describe any methods used to examine subgroups and interactions | In Statistical analyses section, Methods |
|  |  | (*c*) Explain how missing data were addressed | Analyses were based on complete mortality records during the study period. |
|  |  | (*d*) *Cohort study*—If applicable, explain how loss to follow-up was addressed  *Case-control study*—If applicable, explain how matching of cases and controls was addressed  *Cross-sectional study*—If applicable, describe analytical methods taking account of sampling strategy | NA |
|  |  | (*e*) Describe any sensitivity analyses | In the Sensitivity analyses section, Methods. |

| Results | | |  |
| --- | --- | --- | --- |
| Participants | 13* | (a) Report numbers of individuals at each stage of study—eg numbers potentially eligible, examined for eligibility, confirmed eligible, included in the study, completing follow-up, and analysed | In paragraph 1, Results |
|  |  | (b) Give reasons for non-participation at each stage | NA |
|  |  | (c) Consider use of a flow diagram | In S1 Figure. |
| Descriptive data | 14* | (a) Give characteristics of study participants (e.g. demographic, clinical, social) and information on exposures and potential confounders | In paragraph 1, Results |
|  |  | (b) Indicate number of participants with missing data for each variable of interest | Participants with missing data for the interested variables were excluded from the study. |
|  |  | (c) *Cohort study*—Summarise follow-up time (eg, average and total amount) | NA |
| Outcome data | 15* | *Cohort study*—Report numbers of outcome events or summary measures over time | NA |
|  |  | *Case-control study—*Report numbers in each exposure category, or summary measures of exposure | NA |
|  |  | *Cross-sectional study—*Report numbers of outcome events or summary measures | In paragraph 1, Results |
| Main results | 16 | (*a*) Give unadjusted estimates and, if applicable, confounder-adjusted estimates and their precision (eg, 95% confidence interval). Make clear which confounders were adjusted for and why they were included | Throughout the Results Section. |
|  |  | (*b*) Report category boundaries when continuous variables were categorized | NA |
|  |  | (*c*) If relevant, consider translating estimates of relative risk into absolute risk for a meaningful time period | NA |
| Other analyses | 17 | Report other analyses done—e.g. analyses of subgroups and interactions, and sensitivity analyses | In The association between daily PM_2.5_ and YLL section, Results |
| Discussion | | |  |
| Key results | 18 | Summarise key results with reference to study objectives | In paragraph 1, Discussion |
| Limitations | 19 | Discuss limitations of the study, taking into account sources of potential bias or imprecision. Discuss both direction and magnitude of any potential bias | In paragraph 9, Discussion |
| Interpretation | 20 | Give a cautious overall interpretation of results considering objectives, limitations, multiplicity of analyses, results from similar studies, and other relevant evidence | In paragraph 2-8, Discussion |
| Generalisability | 21 | Discuss the generalisability (external validity) of the study results | In paragraph 10, Discussion |
| Other information | | |  |
| Funding | 22 | Give the source of funding and the role of the funders for the present study and, if applicable, for the original study on which the present article is based | In Funding |

*Give information separately for cases and controls in case-control studies and, if applicable, for exposed and unexposed groups in cohort and cross-sectional studies.

**Note:** An Explanation and Elaboration article discusses each checklist item and gives methodological background and published examples of transparent reporting. The STROBE checklist is best used in conjunction with this article (freely available on the Web sites of PLoS Medicine at http://www.plosmedicine.org/, Annals of Internal Medicine at http://www.annals.org/, and Epidemiology at http://www.epidem.com/). Information on the STROBE Initiative is available at www.strobe-statement.org.
