# Supplementary material for: Potential gains in life expectancy by attaining daily ambient fine particulate matter pollution standards in mainland China: A modeling study based on nationwide data
Source: PLoS Med. 2020 Jan 17;17(1):e1003027. doi: 10.1371/journal.pmed.1003027 (PMC6968855; doi:10.1371/journal.pmed.1003027)
Supplement: S1 Table — (DOCX) [file pmed.1003027.s002.docx]

**S1 Table. The list of 72 cities in our study.**

|  | Region | Province | City | Latitude | Longitude |
| --- | --- | --- | --- | --- | --- |
|  | East | Anhui | Bengbu | 33.11037248 | 117.3285674 |
|  | East | Anhui | Huangshan | 29.91097851 | 118.0764606 |
|  | East | Anhui | Ma'anshan | 31.5366855 | 118.6212196 |
|  | East | Anhui | Suzhou | 33.86031277 | 117.2107086 |
|  | East | Anhui | Tongling | 30.94267768 | 117.8851581 |
|  | North | Beijing | Beijing | 40.18695288 | 116.4110932 |
|  | Southwest | Chongqing | Chongqing | 30.05968535 | 107.87553 |
|  | East | Fujian | Nanping | 27.34118149 | 118.1445407 |
|  | Northwest | Gansu | Jinchang | 38.43026804 | 102.0517784 |
|  | Northwest | Gansu | Tianshui | 34.6512979 | 105.7411811 |
|  | Northwest | Gansu | Zhangye | 38.95262888 | 99.85250219 |
|  | South | Guangdong | Guangzhou | 23.35097248 | 113.5372608 |
|  | South | Guangdong | Jiangmen | 22.2820937 | 112.6742817 |
|  | South | Guangdong | Meizhou | 24.20293014 | 116.0798667 |
|  | South | Guangdong | Shaoguan | 24.81843298 | 113.7726202 |
|  | South | Guangxi | Hechi | 24.64377452 | 107.8398216 |
|  | South | Guangxi | Liuzhou | 24.94581318 | 109.3690902 |
|  | South | Guangxi | Nanning | 23.05811763 | 108.4652859 |
|  | South | Guangxi | Qinzhou | 22.1874002 | 109.0289448 |
|  | Southwest | Guizhou | Liupanshui | 26.13595429 | 104.8890978 |
|  | Southwest | Guizhou | Zunyi | 28.16859749 | 107.0868008 |
|  | North | Hebei | Cangzhou | 38.2673799 | 116.7459782 |
|  | North | Hebei | Xingtai | 37.21268069 | 114.8194542 |
|  | Northeast | Heilongjiang | Harbin | 45.63756951 | 127.9569287 |
|  | Central | Henan | Anyang | 35.88059171 | 114.3501897 |
|  | Central | Henan | Xinxiang | 35.26499853 | 114.0915639 |
|  | Central | Hubei | Enshi | 30.19249008 | 109.5498173 |
|  | Central | Hubei | Huanggang | 30.71717017 | 115.3397949 |
|  | Central | Hubei | Yichang | 30.74985171 | 111.1377138 |
|  | Central | Hunan | Chenzhou | 25.81333594 | 113.1360848 |
|  | Central | Hunan | Huaihua | 27.54796973 | 110.0743627 |
|  | Central | Hunan | Changsha | 28.22887147 | 113.1524111 |
|  | East | Jiangsu | Changzhou | 31.63012132 | 119.634944 |
|  | East | Jiangsu | Lianyungang | 34.53481763 | 119.1328506 |
|  | East | Jiangsu | Taizhou | 32.57267207 | 120.0561648 |
|  | East | Jiangsu | Wuxi | 31.52685876 | 120.0770537 |
|  | East | Jiangsu | Xuzhou | 34.36073168 | 117.5127239 |
|  | East | Jiangsu | Yangzhou | 32.73928264 | 119.4728542 |
|  | East | Jiangxi | Ganzhou | 25.70827198 | 115.2724875 |
|  | East | Jiangxi | Xinyu | 27.84797879 | 114.8520244 |
|  | Northeast | Jilin | Jilin | 43.58071226 | 126.8443023 |
|  | Northeast | Jilin | Yanbian | 43.14479774 | 129.1275139 |
|  | Northeast | Liaoning | Benxi | 41.23034338 | 124.5664724 |
|  | Northeast | Liaoning | Jinzhou | 41.47818111 | 121.6225153 |
|  | Northeast | Liaoning | Shenyang | 42.09768454 | 123.1371311 |
|  | Northeast | Liaoning | Tieling | 42.64368007 | 124.1703741 |
|  | Northwest | Ningxia | Shizuishan | 39.01235979 | 106.5140045 |
|  | Northwest | Qinghai | Huangnan | 35.02907233 | 101.6313118 |
|  | Northwest | Shaanxi | Baoji | 34.37980922 | 107.2031598 |
|  | East | Shandong | Heze | 35.15579967 | 115.6921261 |
|  | East | Shandong | Jinan | 36.73835363 | 117.0920564 |
|  | East | Shandong | Weihai | 37.12465083 | 121.9813021 |
|  | East | Shandong | Weifang | 36.54039692 | 119.0769118 |
|  | East | Shandong | Yantai | 37.24631023 | 120.8073299 |
|  | East | Shandong | Zaozhuang | 34.91917688 | 117.3952413 |
|  | East | Shandong | Zibo | 36.60944072 | 118.057108 |
|  | East | Shanghai | Shanghai | 31.18546101 | 121.4208458 |
|  | North | Shanxi | Jinzhong | 37.3290385 | 112.9639443 |
|  | North | Shanxi | Taiyuan | 37.96112892 | 112.3150466 |
|  | Southwest | Sichuan | Guangyuan | 32.26211873 | 105.7797223 |
|  | Southwest | Sichuan | Panzhihua | 26.80752596 | 101.7312873 |
|  | Southwest | Sichuan | Zigong | 29.28740643 | 104.6827647 |
|  | North | Tianjin | Tianjin | 39.31417415 | 117.3249732 |
|  | Southwest | Tibet | Lhasa | 30.0348938 | 91.09014949 |
|  | Southwest | Tibet | Naqu | 32.88196168 | 89.0347145 |
|  | Southwest | Tibet | Shannan | 28.29975925 | 92.26311112 |
|  | Northwest | Xinjiang | Hami | 43.04140931 | 93.51352336 |
|  | Northwest | Xinjiang | Kezi | 39.70203691 | 75.98811184 |
|  | Southwest | Yunnan | Baoshan | 24.97603196 | 98.97450111 |
|  | Southwest | Yunnan | Yuxi | 24.13864646 | 102.1997581 |
|  | East | Zhejiang | Jinhua | 29.11688048 | 119.954105 |
|  | East | Zhejiang | Shaoxing | 29.7163403 | 120.6319989 |
