# Supplementary material for: Potential gains in life expectancy by attaining daily ambient fine particulate matter pollution standards in mainland China: A modeling study based on nationwide data
Source: PLoS Med. 2020 Jan 17;17(1):e1003027. doi: 10.1371/journal.pmed.1003027 (PMC6968855; doi:10.1371/journal.pmed.1003027)
Supplement: S2 Table — (DOCX) [file pmed.1003027.s003.docx]

**S2 Table. The list of model parameters in this study.**

| **Parameter** | **Range** | **Caveats** |
| --- | --- | --- |
| Years of life lost | 2.4 to 3021.5 | Dependent variable |
| PM_2.5_ (μg/m^3^) | 3.6 to 985.2 |  |
| Long-term trend | 1 to 1461 | Degrees of freedom = 6 per year |
| Day of the week | 1 to 7 |  |
| Public holiday | 0 and 1 |  |
| Temperature (°C) | -28.0 to 36.5 | Degrees of freedom = 6 |
| Relative humidity (%) | 5.0 to 100.0 | Degrees of freedom = 3 |

Abbreviations: PM_2.5_ = particulate matter with an aerodynamic diameter less than or equal to 2.5 μm.
