# Supplementary material for: Potential gains in life expectancy by attaining daily ambient fine particulate matter pollution standards in mainland China: A modeling study based on nationwide data
Source: PLoS Med. 2020 Jan 17;17(1):e1003027. doi: 10.1371/journal.pmed.1003027 (PMC6968855; doi:10.1371/journal.pmed.1003027)
Supplement: S3 Table — (DOCX) [file pmed.1003027.s004.docx]

**S3 Table. Spearman correlation between air pollutants and meteorological factors in 72 cities of mainland China, 2013–2016.**

|  | PM_2.5_ | SO_2_ | NO_2_ | O_3_ | Mean temperature | Relative humidity |
| --- | --- | --- | --- | --- | --- | --- |
| PM_2.5_ | 1.00 |  |  |  |  |  |
| SO_2_ | 0.26 | 1.00 |  |  |  |  |
| NO_2_ | 0.50 | 0.37 | 1.00 |  |  |  |
| O_3_ | 0.29 | 0.36 | -0.02 | 1.00 |  |  |
| Mean temperature | -0.15 | -0.33 | -0.30 | 0.25 | 1.00 |  |
| Relative humidity | -0.02 | -0.12 | -0.06 | -0.16 | 0.24 | 1.00 |

Abbreviations: PM_2.5_=particulate matter with an aerodynamic diameter less than or equal to 2.5 μm; SO_2_=sulfur dioxide; NO_2_=nitrogen dioxide; O_3_=ozone.
