# Supplementary material for: Potential gains in life expectancy by attaining daily ambient fine particulate matter pollution standards in mainland China: A modeling study based on nationwide data
Source: PLoS Med. 2020 Jan 17;17(1):e1003027. doi: 10.1371/journal.pmed.1003027 (PMC6968855; doi:10.1371/journal.pmed.1003027)
Supplement: S4 Table — PM2.5, particulate matter with an aerodynamic diameter less than or equal to 2.5 μm or fine particulate matter; YLL, years of life lost. (DOCX) [file pmed.1003027.s005.docx]

**S4 Table. Regional-specific estimates of absolute change in YLL associated each 10 μg/m^3^ increase in PM_2.5_ in single- and two-pollutant models in 72 cities of mainland China, 2013–2016.**

| Pollutants and Models | YLL (95% CI) ^a^ |
| --- | --- |
| Northwest |  |
| Single-pollutant model | 0.94 (0.21, 1.68) |
| + SO_2_ | 0.89 (0.24, 1.54) |
| + NO_2_ | 0.85 (0.03, 1.68) |
| + O_3_ | 0.90 (0.11, 1.69) |
| North |  |
| Single-pollutant model | 0.12 (0.03, 0.22) |
| + SO_2_ | 0.12 (0.03, 0.22) |
| + NO_2_ | 0.13 (0.03, 0.23) |
| + O_3_ | 0.12 (0.02, 0.22) |
| Northeast |  |
| Single-pollutant model | 0.43 (0.05, 0.81) |
| + SO_2_ | 0.24 (-0.02, 0.51) |
| + NO_2_ | 0.25 (-0.04, 0.54) |
| + O_3_ | 0.43 (0.03, 0.83) |
| Central |  |
| Single-pollutant model | 0.61 (0.05, 1.17) |
| + SO_2_ | 0.61 (0.10, 1.11) |
| + NO_2_ | 0.51 (0.04, 0.98) |
| + O_3_ | 0.65 (0.09, 1.20) |
| East |  |
| Single-pollutant model | 0.37 (0.13, 0.61) |
| + SO_2_ | 0.23 (0.05, 0.41) |
| + NO_2_ | 0.27 (0.02, 0.52) |
| + O_3_ | 0.30 (0.06, 0.53) |
| Southwest |  |
| Single-pollutant model | 0.85 (0.60, 1.09) |
| + SO_2_ | 1.00 (0.68, 1.33) |
| + NO_2_ | 0.71 (0.48, 0.94) |
| + O_3_ | 0.86 (0.57, 1.16) |
| South |  |
| Single-pollutant model | 0.58 (0.25, 0.92) |
| + SO_2_ | 0.62 (0.24, 1.00) |
| + NO_2_ | 0.37 (0.01, 0.73) |
| + O_3_ | 0.49 (0.14, 0.84) |
| Overall effect estimates (pooled estimate) |  |
| Single-pollutant model | 0.43 (0.29, 0.57) |
| + SO_2_ | 0.41 (0.27, 0.55) |
| + NO_2_ | 0.32 (0.19, 0.45) |
| + O_3_ | 0.41 (0.27, 0.55) |

^#:^ Moving averaged concentration of lag 0 to lag 2 of daily PM_2.5_;

^a:^ Absolute change in years of life lost;

Abbreviations: PM_2.5_=particulate matter with an aerodynamic diameter less than or equal to 2.5 μm; SO_2_=sulfur dioxide; NO_2_=nitrogen dioxide; O_3_=ozone; YLL=years of life lost; CI, confidence interval.
