# Supplementary material for: Potential gains in life expectancy by attaining daily ambient fine particulate matter pollution standards in mainland China: A modeling study based on nationwide data
Source: PLoS Med. 2020 Jan 17;17(1):e1003027. doi: 10.1371/journal.pmed.1003027 (PMC6968855; doi:10.1371/journal.pmed.1003027)
Supplement: S5 Table — PM2.5, particulate matter with an aerodynamic diameter less than or equal to 2.5 μm or fine particulate matter; YLL, years of life lost. (DOCX) [file pmed.1003027.s006.docx]

**S5 Table. Sensitivity analyses for the absolute change in YLL associated with each 10 μg/m^3^ increase in PM_2.5_ in different models.**

| **Regions** | **Model 1** | **Model 2** |
| --- | --- | --- |
| Northwest | 0.94 (0.21, 1.68) | 0.76 (0.35, 1.18) |
| North | 0.12 (0.03, 0.22) | 0.08 (-0.04, 0.19) |
| Northeast | 0.43 (0.05, 0.81) | 0.43 (0.22, 0.64) |
| Central | 0.61 (0.05, 1.17) | 0.23 (-0.12, 0.58) |
| East | 0.37 (0.13, 0.61) | 0.33 (0.15, 0.52) |
| Southwest | 0.85 (0.60, 1.09) | 0.68 (0.44, 0.93) |
| South | 0.58 (0.25, 0.92) | -0.07 (-0.47, 0.33) |
| National | 0.43 (0.29, 0.57) | 0.18 (0.09, 0.27) |

Model 1: Two-stage model which generating the regional and national estimates by meta-analysis;

Model 2: Mix-effects regression model adjusting for the variable of city with a random term.
