# Supplementary material for: Potential gains in life expectancy by attaining daily ambient fine particulate matter pollution standards in mainland China: A modeling study based on nationwide data
Source: PLoS Med. 2020 Jan 17;17(1):e1003027. doi: 10.1371/journal.pmed.1003027 (PMC6968855; doi:10.1371/journal.pmed.1003027)
Supplement: S6 Table — PM2.5, particulate matter with an aerodynamic diameter less than or equal to 2.5 μm or fine particulate matter; YLL, years of life lost. (DOCX) [file pmed.1003027.s007.docx]

**S6 Table. Change in PM_2.5_–YLL relationship per IQR increase in city-level variables.**

| City-level variables | IQR | Change in PM_2.5_–YLL relationship (%) | *P*-value |
| --- | --- | --- | --- |
| Gross Domestic Product (in billions, CNY) | 4.98 | 0.10 (-0.22, 0.41) | 0.55 |
| Population density (in millions) | 102.65 | 0.00 (-0.02, 0.03) | 0.81 |
| GDP per capita (in billions, CNY) | 0.025 | 0.02 (-0.05, 0.05) | 0.97 |
| Elevation (m) | 944.70 | -0.32 (-0.97, 0.34) | 0.34 |
| Precipitation (mm) | 66.97 | -0.35 (-0.97, 0.27) | 0.27 |
| Poverty rate (%) | 20.99 | -0.04 (-0.28, 0.2) | 0.74 |
| Education level (by year of school) | 1.71 | -0.14 (-0.46, 0.18) | 0.40 |
| Annual PM_2.5_ concentration (μg/m^3^) | 39.40 | -0.59 (-1.18, 0.00) | <0.05 |
| Annual CO concentration (μg/m^3^) | 129.24 | 0.11 (-0.33, 0.56) | 0.62 |
| Annual O_3_ concentration (μg/m^3^) | 22.58 | -0.34 (-0.73, 0.05) | 0.09 |
| Annual SO_2_ concentration (μg/m^3^) | 25.61 | -0.01 (-0.31, 0.29) | 0.94 |
| Annual NO_2_ concentration (μg/m^3^) | 16.12 | -0.01 (-0.46, 0.43) | 0.95 |
| Annual air pressure (kPa) | 69.42 | -0.11 (-0.66, 0.45) | 0.70 |
| Annual temperature (℃) | 6.33 | 0.27 (-0.1, 0.64) | 0.15 |
| Annual relative humidity (%) | 18.06 | -0.11 (-0.68, 0.47) | 0.72 |

Abbreviations: PM_2.5_ = particulate matter with an aerodynamic diameter less than or equal to 2.5 μm; YLL = years of life lost; IQR = the difference between the third quartile and the first quartile; CNY = Chinese Yuan; CO = carbon monoxide; O_3_ = ozone; SO_2_ = sulfur dioxide; NO_2_ = nitrogen dioxide.
