# Supplementary material for: Potential gains in life expectancy by attaining daily ambient fine particulate matter pollution standards in mainland China: A modeling study based on nationwide data
Source: PLoS Med. 2020 Jan 17;17(1):e1003027. doi: 10.1371/journal.pmed.1003027 (PMC6968855; doi:10.1371/journal.pmed.1003027)
Supplement: S1 Text — (DOCX) [file pmed.1003027.s008.docx]

**S1 Text. Prospective analysis plan of the current study.**

**Period of this research:** July 2016 through June 2020

**Research type:** A nationwide modelling study based on a time series analysis

**Research objectives:**

1. To examine the associations between ambient PM_2.5_ and years of life lost (YLL) at both national and regional levels in mainland China.

2. To estimate the potential gains in life expectancy (PGLE) by postulating that ambient PM_2.5_ concentrations were controlled under the Chinese National Ambient Air Quality Standards (NAAQS), as well as the World Health Organization’s (WHO’s) air quality guideline **(**AQG) and its Interim Targets (ITs) in 72 cities across mainland China.

3. To estimate the proportion of YLL attributable to ambient PM_2.5_ exposure in 72 cities across mainland China.

**Subjects:**

Our data consist of daily count of non-accidental mortality and daily YLL for the period between 2013 and 2016 in 72 cities across mainland China. The data will be obtained from the Death Surveillance Points System of China.

**PM_2.5_ measurements:**

Daily concentrations for ambient PM_2.5_ and other ambient air pollutants will be obtained from the China’s National Real-time Publishing Platform for Urban Air Quality (<http://106.37.208.233:20035>).

**Indicators:**

1. We will use the age-specific life expectancy in the corresponding years to calculate the YLL for each death by matching age and sex to the Chinese national life table.

2. The PGLE denotes the average gains in life expectancy for each death that can be prevented by attaining the ambient air quality standard/guidelines for PM_2.5_, which can be calculated by the following formulas:

$$PGLE=\frac{Avoidable YLLs}{Over mortality count}$$

where the avoidable YLL is the sum of years of life lost that can be prevented in the study area by controlling the ambient PM_2.5_ concentration; the overall mortality count is the total mortality count during the study period.

**Analyses:**

**1. Descriptive analyses**

We will perform a descriptive analysis to summarize the number of cities, daily mortality and YLL, air pollutants, temperature, and relative humidity in each region during the study period. The Spearman correlation will be utilized to quantify the correlation between air pollutants and weather conditions.

**2. The association between PM_2.5_ and YLL**

A two-stage hierarchical approach will be applied to examine the national and regional association between PM_2.5_ and YLL. In the first stage, we will apply a generalized additive model (GAM) with a Gaussian link to explore the city-specific short-term association between PM_2.5_ and YLL. In the second stage, we will use random-effects meta-analysis to generate both regional and national estimates, and both an I^2^ statistic and a Q-test will be used to test the variability of the estimated effects between different cities.

**3. Estimating potential gains in life expectancy by controlling PM_2.5_ concentrations**

Based on the established associations between ambient PM_2.5_ and YLL, we will further estimate the avoidable YLL and PGLE by assuming the ambient PM_2.5_ had been controlled at specified concentrations by the Chinese NAAQS or WHO’s AQG and its ITs.

**4. Sensitivity analyses**

Sensitivity analyses will be performed to test the robustness of our findings. Two-pollutant models will be conducted to examine the robustness of associations between daily PM_2.5_ and YLL. PM_2.5_ will be included alone in the single-pollutant models, while SO_2_ (or NO_2_, O_3_) and PM_2.5_ will be included jointly in two-pollutant models.
