# Supplementary figures and images for: Potential gains in life expectancy by attaining daily ambient fine particulate matter pollution standards in mainland China: A modeling study based on nationwide data
Source: PLoS Med. 2020 Jan 17;17(1):e1003027. doi: 10.1371/journal.pmed.1003027 (PMC6968855; doi:10.1371/journal.pmed.1003027)

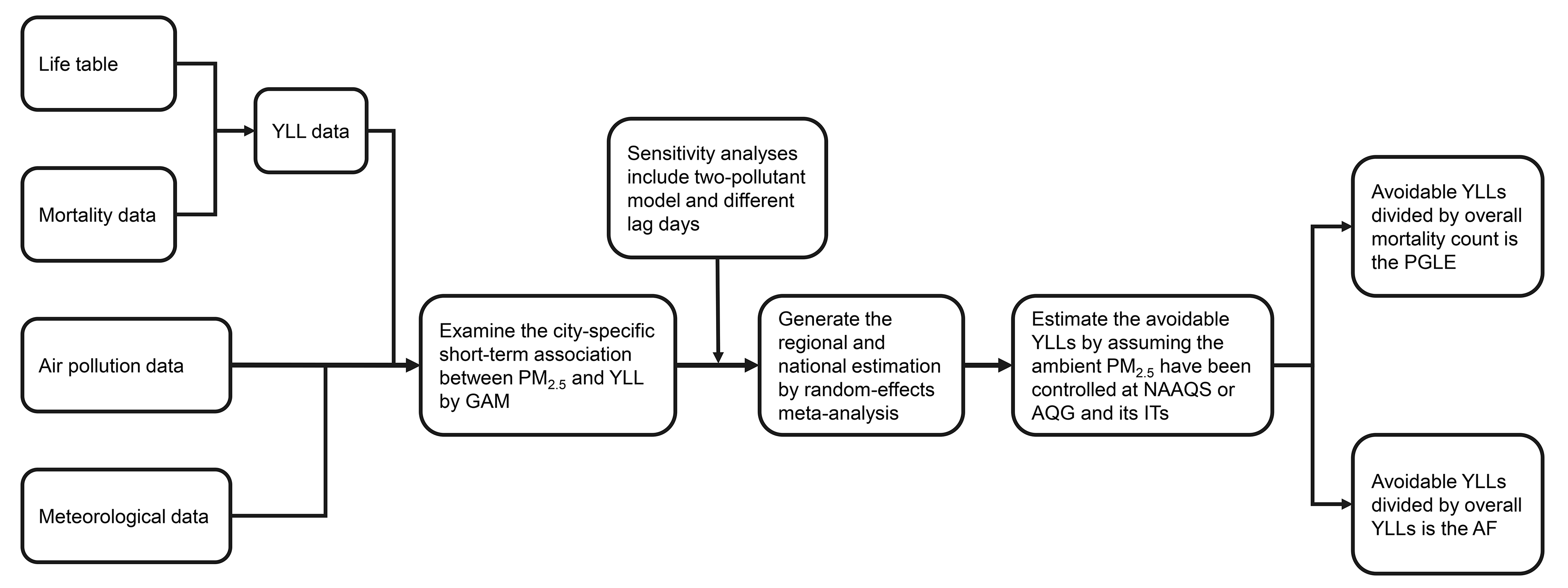

Supplement: S1 Fig — (TIF) [file pmed.1003027.s009.tif]

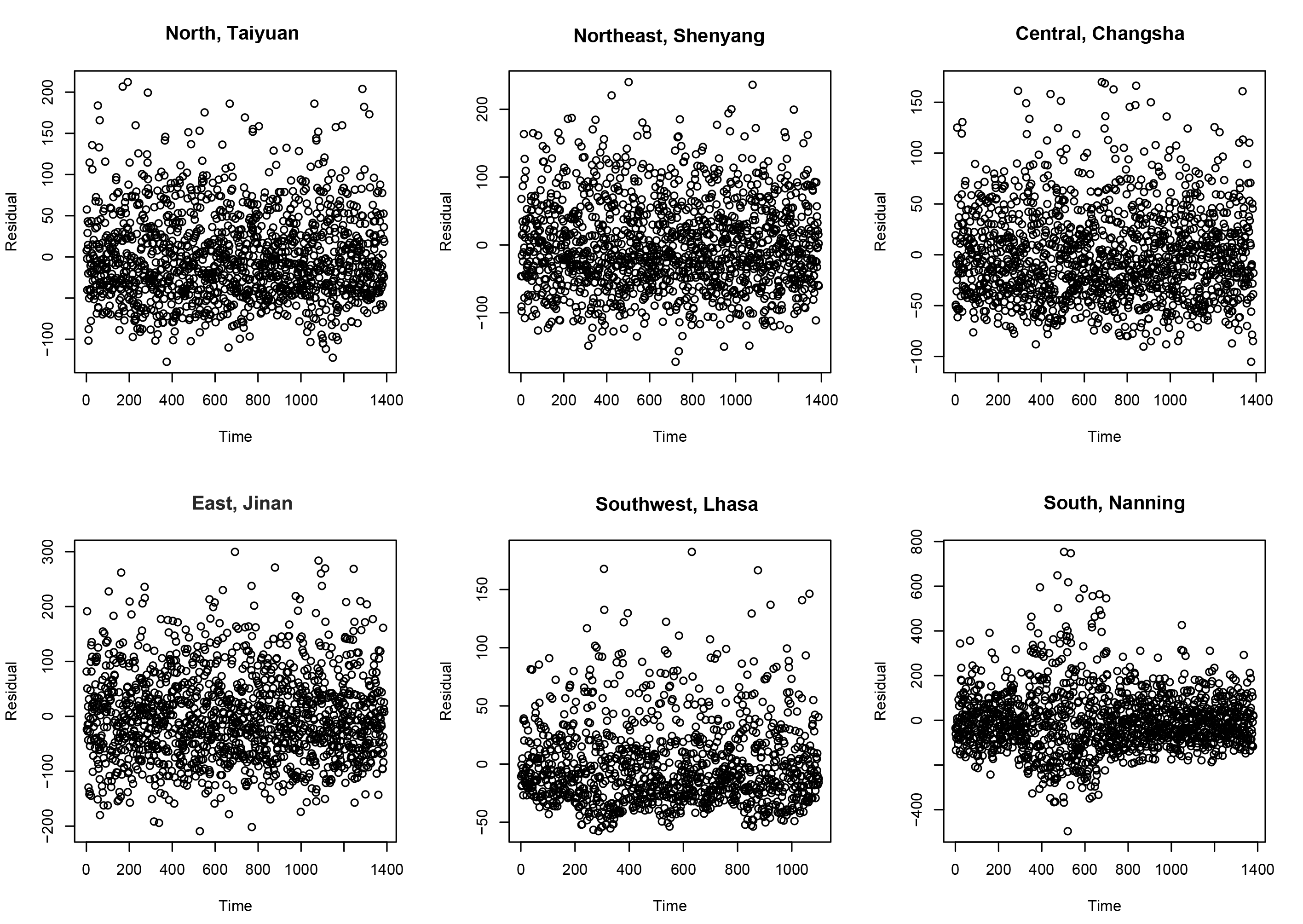

Supplement: S2 Fig — (TIF) [file pmed.1003027.s010.tif]

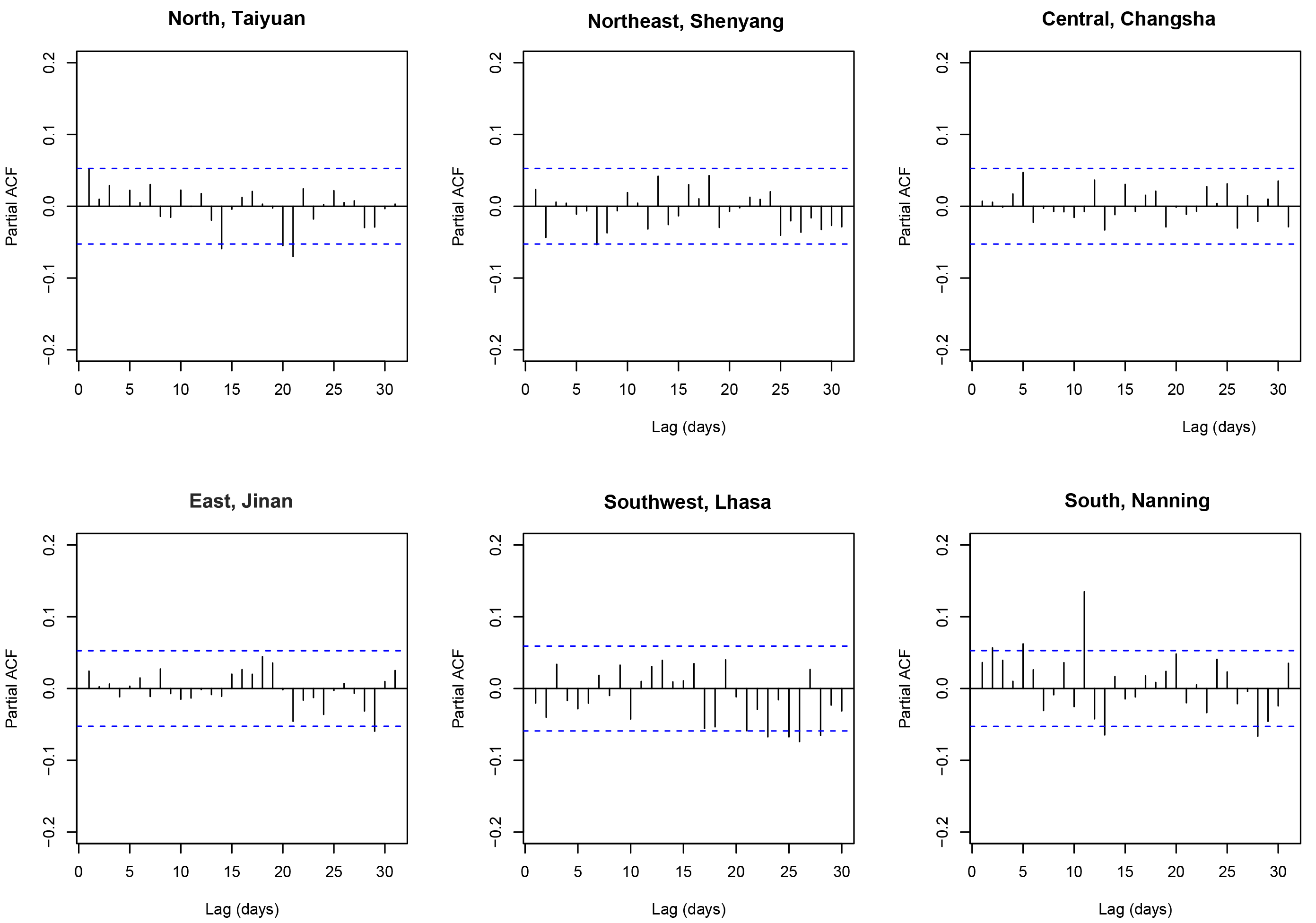

Supplement: S3 Fig — PACF, partial autocorrelation function. (TIF) [file pmed.1003027.s011.tif]

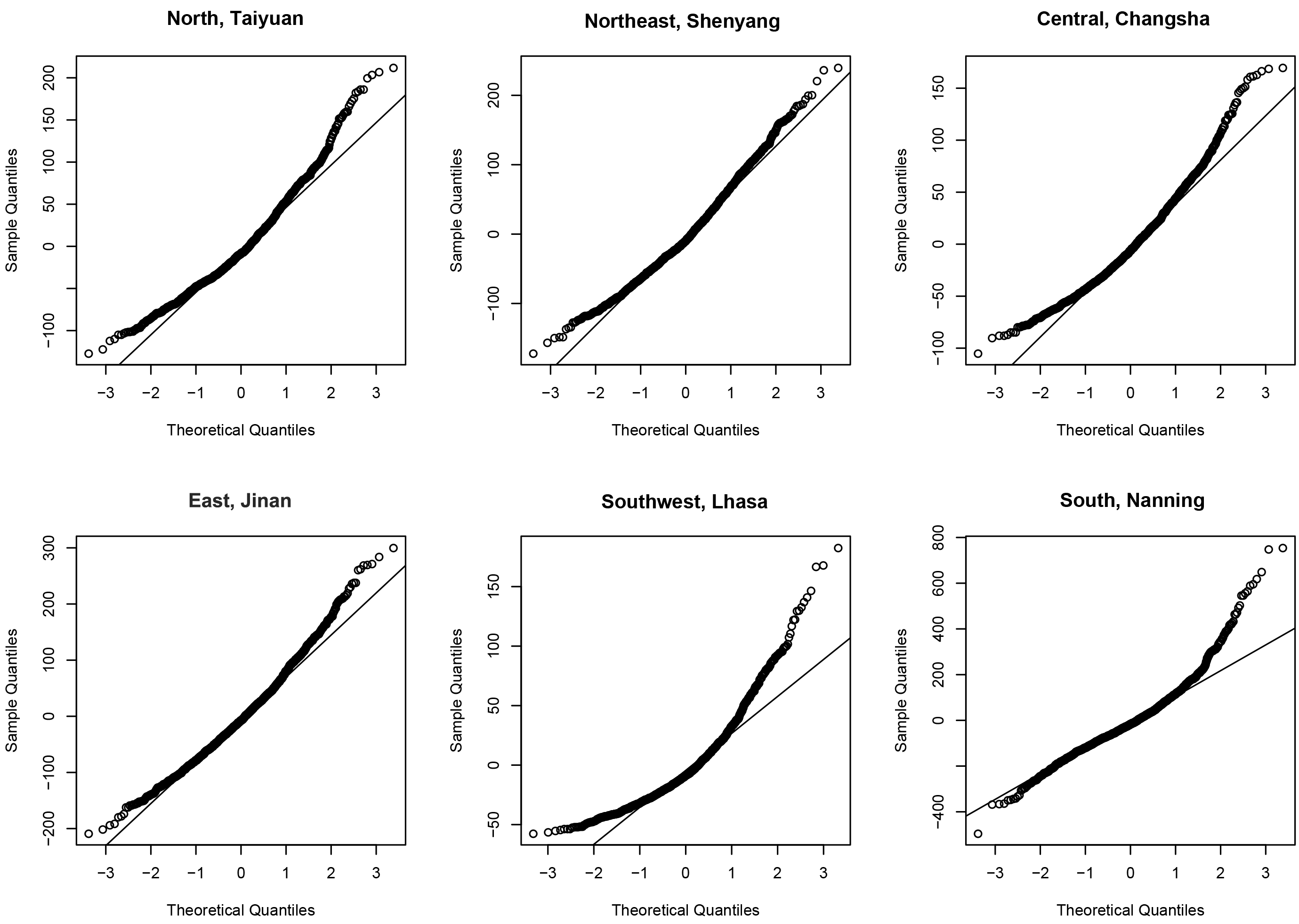

Supplement: S4 Fig — (TIF) [file pmed.1003027.s012.tif]

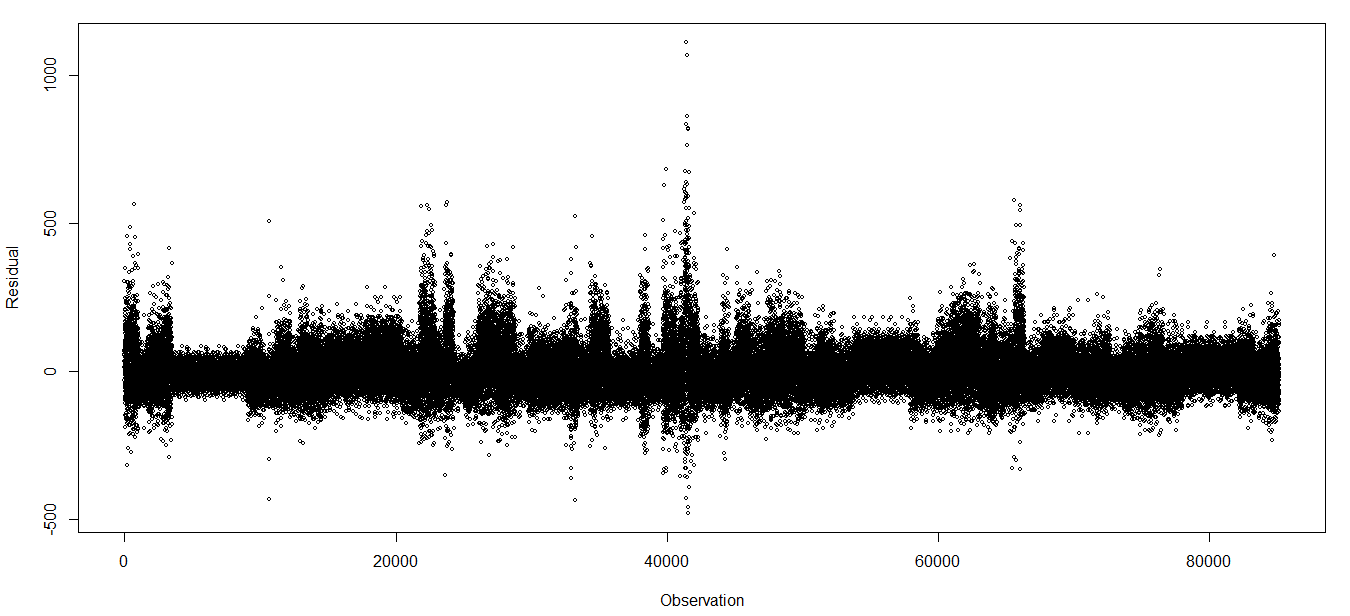

Supplement: S5 Fig — (TIF) [file pmed.1003027.s013.tif]
