# Supplementary material for: Potential gains in life expectancy by attaining daily ambient fine particulate matter pollution standards in mainland China: A modeling study based on nationwide data
Source: PLoS Med. 2020 Jan 17;17(1):e1003027. doi: 10.1371/journal.pmed.1003027 (PMC6968855; doi:10.1371/journal.pmed.1003027)
Supplement: Attachment — Submitted filename: 1 Response_20190926v6.docx [file pmed.1003027.s014.docx]

**Response to the comments from the editor and reviewers**

Dear Editor,

We are grateful for the careful reviews and insightful comments on our manuscript. We have extensively revised the manuscript accordingly. The specific point-to-point responses are shown below.

**Requests from the editors:**

1- Did your study have a prospective protocol or analysis plan? Please state this (either way) early in the Methods section.

a) If a prospective analysis plan (from your funding proposal, IRB or other ethics committee submission, study protocol, or other planning document written before analyzing the data) was used in designing the study, please include the relevant prospectively written document with your revised manuscript as a Supporting Information file to be published alongside your study, and cite it in the Methods section. A legend for this file should be included at the end of your manuscript.

b) If no such document exists, please make sure that the Methods section transparently describes when analyses were planned, and when/why any data-driven changes to analyses took place.

c) In either case, changes in the analysis—including those made in response to peer review comments—should be identified as such in the Methods section of the paper, with rationale.

**Response:** Yes, we do have a prospective protocol and this study was supported by the National Key R&D Program of China (2016YFC0206501). The specific protocol has been included in a supplementary file (S3 Text).

In the **Methods** section, we added “The data analyses were performed following a prospective analysis plan (S3 Text)”.

2- Thank you for providing information on how readers can access the primary data. In addition to the url for the air pollutant concentrations, please include the url or email contact information for obtaining meteorological data from the National Weather Data Sharing System of China. Additionally, while we understand that you cannot share the daily mortality time series data, please provide contact information (url or email) you used to obtain this data from the Death Surveillance Points System of China.

**Response:** Thanks for raising this important issue. As suggested, we have included the URL for obtaining meteorological data from the National Weather Data Sharing System of China and mortality data from the Death Surveillance Points System of China in the revised manuscript.

That sentence has been changed to: “The daily time-series mortality data on non-accidental causes in 72 Chinese cities (S1 Table) for the period of January 18, 2013 through December 31, 2016 were selected for this study, and a total of 1,226,849 non-accidental deaths were recorded. The data were extracted from the Death Surveillance Points System (DSP) of China, which is operated by the National Center for Chronic and Noncommunicable Disease Control and Prevention, Chinese Center for Disease Control and Prevention (official contact email: syjc@ncncd.chinacdc.cn) [1]” **and** “In addition, daily meteorological data on mean temperature (°C) and relative humidity (%) were obtained from the National Meteorological Data Service Center of China (http://data.cma.cn), which are publicly accessible.”.

3- Please place the study design in your title following the colon. We suggest: “Potential gains in life expectancy by attaining daily ambient fine particulate matter pollution standards in mainland China: a modelling study based on nationwide data”.

**Response:** Done as suggested. The title has been revised to “Potential gains in life expectancy by attaining daily ambient fine particulate matter pollution standards in mainland China: a modelling study based on nationwide data”.

4- Please structure your abstract using the PLOS Medicine headings (Background, Methods and Findings, Conclusions).

**Response:** Thanks. We have re-structured the Abstract according to the PLOS Medicine format. Please see the revised manuscript.

5- Please provide the context of why the study is important in the Abstract Background. The final sentence should clearly state the study question.

**Response:** Done as suggested. The **Abstract Background** has been revised to: “Ambient fine particulate matter pollution (PM_2.5_) is one leading cause of disease burden, but no study has quantified the association between daily PM_2.5_ exposure and life expectancy. We aimed to assess the potential benefits in life expectancy by attaining the daily PM_2.5_ standards in China during 2013-2016.”.

6- In the Abstract Methods and Findings, please include the total population of the 72 cities, and the exact date range (include day and month) of the study.

**Response:** Thank you for raising these issues. Accordingly, we have included the total population and exact date range of the study in the **Abstract Methods and Findings**: “A total of 72 cities were included in this study with an overall population of 495.93 million.” **and** “During the period from January 18, 2013 to December 31, 2016, we recorded 1,226,849 non-accidental deaths in the study area”.

7- It would also be helpful to include the average life expectancy and other demographic information (average age, % female) of the population included.

**Response:** Thanks for the insightful suggestion. We have added the above-mentioned information in the revised manuscript: “During the period from January 18, 2013 to December 31, 2016, we recorded 1,226,849 non-accidental deaths in the study area; 44.0% of the study population were females. The average life expectancy in China was 76.25 years in 2016, and the average death age of the subjects included in our study was 71.72±16.74 years.”

8- In the last sentence of the Abstract Methods and Findings section, please describe the main limitation(s) of the study's methodology.

**Response:** Done. We have made corrections accordingly. In the last sentence of the **Abstract Methods and Findings** section, we added: “Findings from this study were mainly limited by the unavailability of data on individual PM_2.5_ exposure.”.

9- Is it worth specifying in the Abstract Conclusions that these estimates and conclusions are based on short-term air pollution exposure?

**Response:** We appreciate the valuable comment. A few previous studies have reported the effects of long-term air pollution exposure on life expectancy, but little is known about the effect on life expectancy due to short-term air pollution exposure. For this reason, we prefer to keep the statement.

10- At this stage, we ask that you include a short, non-technical Author Summary of your research to make findings accessible to a wide audience that includes both scientists and non-scientists. The Author Summary should immediately follow the Abstract in your revised manuscript. This text is subject to editorial change and should be distinct from the scientific abstract. Please see our author guidelines for more information: <https://journals.plos.org/plosmedicine/s/revising-your-manuscript#loc-author-summary>

**Response:** Many thanks. We have added an Author Summary of this research immediately following the Abstract in the revised manuscript as the following:

**Author summary**

**Why was this study done?**

- Ambient fine particulate matter (PM_2.5_) pollution is a severe environmental health concern in China.
- Both short-term and long-term exposures to PM_2.5_ pollution have been found to be associated with increased mortality and years of life lost.
- A few studies have estimated the association between annual PM_2.5_ concentration and life expectancy, but there is no report on the effects of daily PM_2.5_ exposure on life expectancy.

**What did the researchers do and find?**

- This nationwide time-series study collected data on more than 1 million non-accidental deaths in 72 cities in China from January 18, 2013 to December 31, 2016.
- We used a generalized additive model to explore the city-specific association between daily PM_2.5_ and years of life lost and then conducted random-effects meta-analyses to generate the regional and national estimates.
- We estimated that 168065.18 years of life lost can be avoided by achieving the WHO’s guideline on daily PM_2.5_ concentrations in the study area, which corresponded to 0.14 years of gains in life expectancy for each death in the study area.
- By adopting the WHO’s guideline as a reference, we estimated that about 1.00% of the years of life lost could be avoided in the study area.

**What do these findings mean?**

- This is the first study to report the potential gains in life expectancy by attaining the daily standards of PM_2.5_, which provided important and useful information of the burden caused by ambient PM_2.5_ pollution.
- In the future, there should be a policy of stricter ambient PM_2.5_ standards at both nationally and on regional levels in China, which would benefit the population’s health and life expectancy.

11- Please provide the name(s) of the institutional review board(s) that provided ethical approval, and whether informed consent was provided or waived (if waived, by whom).

**Response:** Added as suggested. This study was based on the National Key R&D Program of China (2016YFC0206501), and this project aims to examine the short-term health effects of air pollution in China, which has been approved by the Ethical Review Committee of Institute for Environmental Health and Related Product Safety, Chinese Center for Disease Control and Prevention. Moreover, the mortality data of our study were collected as part of government-mandated health surveillance and analyzed anonymously. The Committee acknowledged that the rights and the welfare of the subject are adequately protected in our study, so personal consent was not needed. We have added this information in the text: “This study was based on one project aiming to examine the short-term health effects of air pollution in China, which has been approved by the Ethical Review Committee of Institute for Environmental Health and Related Product Safety, Chinese Center for Disease Control and Prevention. And no individual consent was required as all the data were analyzed at aggregated level. The present study is reported as per the Strengthening the Reporting of Observational Studies in Epidemiology (STROBE) guidelines (S2 Table).”.

12- Please include a list of the 72 cities included, perhaps as a supplemental text referenced from the Methods.

**Response:** Thanks for the thoughtful suggestion. We have included a list of the research cities as a supplemental table referenced from the Methods (please see S1 Table).

**S1 Table. The list of 72 cities in our study.**

|  | Region | Province | City | Latitude | Longitude |
| --- | --- | --- | --- | --- | --- |
|  | East | Anhui | Bengbu | 33.11037248 | 117.3285674 |
|  | East | Anhui | Huangshan | 29.91097851 | 118.0764606 |
|  | East | Anhui | Ma'anshan | 31.5366855 | 118.6212196 |
|  | East | Anhui | Suzhou | 33.86031277 | 117.2107086 |
|  | East | Anhui | Tongling | 30.94267768 | 117.8851581 |
|  | North | Beijing | Beijing | 40.18695288 | 116.4110932 |
|  | Southwest | Chongqing | Chongqing | 30.05968535 | 107.87553 |
|  | East | Fujian | Nanping | 27.34118149 | 118.1445407 |
|  | Northwest | Gansu | Jinchang | 38.43026804 | 102.0517784 |
|  | Northwest | Gansu | Tianshui | 34.6512979 | 105.7411811 |
|  | Northwest | Gansu | Zhangye | 38.95262888 | 99.85250219 |
|  | South | Guangdong | Guangzhou | 23.35097248 | 113.5372608 |
|  | South | Guangdong | Jiangmen | 22.2820937 | 112.6742817 |
|  | South | Guangdong | Meizhou | 24.20293014 | 116.0798667 |
|  | South | Guangdong | Shaoguan | 24.81843298 | 113.7726202 |
|  | South | Guangxi | Hechi | 24.64377452 | 107.8398216 |
|  | South | Guangxi | Liuzhou | 24.94581318 | 109.3690902 |
|  | South | Guangxi | Nanning | 23.05811763 | 108.4652859 |
|  | South | Guangxi | Qinzhou | 22.1874002 | 109.0289448 |
|  | Southwest | Guizhou | Liupanshui | 26.13595429 | 104.8890978 |
|  | Southwest | Guizhou | Zunyi | 28.16859749 | 107.0868008 |
|  | North | Hebei | Cangzhou | 38.2673799 | 116.7459782 |
|  | North | Hebei | Xingtai | 37.21268069 | 114.8194542 |
|  | Northeast | Heilongjiang | Harbin | 45.63756951 | 127.9569287 |
|  | Central | Henan | Anyang | 35.88059171 | 114.3501897 |
|  | Central | Henan | Xinxiang | 35.26499853 | 114.0915639 |
|  | Central | Hubei | Enshi | 30.19249008 | 109.5498173 |
|  | Central | Hubei | Huanggang | 30.71717017 | 115.3397949 |
|  | Central | Hubei | Yichang | 30.74985171 | 111.1377138 |
|  | Central | Hunan | Chenzhou | 25.81333594 | 113.1360848 |
|  | Central | Hunan | Huaihua | 27.54796973 | 110.0743627 |
|  | Central | Hunan | Changsha | 28.22887147 | 113.1524111 |
|  | East | Jiangsu | Changzhou | 31.63012132 | 119.634944 |
|  | East | Jiangsu | Lianyungang | 34.53481763 | 119.1328506 |
|  | East | Jiangsu | Taizhou | 32.57267207 | 120.0561648 |
|  | East | Jiangsu | Wuxi | 31.52685876 | 120.0770537 |
|  | East | Jiangsu | Xuzhou | 34.36073168 | 117.5127239 |
|  | East | Jiangsu | Yangzhou | 32.73928264 | 119.4728542 |
|  | East | Jiangxi | Ganzhou | 25.70827198 | 115.2724875 |
|  | East | Jiangxi | Xinyu | 27.84797879 | 114.8520244 |
|  | Northeast | Jilin | Jilin | 43.58071226 | 126.8443023 |
|  | Northeast | Jilin | Yanbian | 43.14479774 | 129.1275139 |
|  | Northeast | Liaoning | Benxi | 41.23034338 | 124.5664724 |
|  | Northeast | Liaoning | Jinzhou | 41.47818111 | 121.6225153 |
|  | Northeast | Liaoning | Shenyang | 42.09768454 | 123.1371311 |
|  | Northeast | Liaoning | Tieling | 42.64368007 | 124.1703741 |
|  | Northwest | Ningxia | Shizuishan | 39.01235979 | 106.5140045 |
|  | Northwest | Qinghai | Huangnan | 35.02907233 | 101.6313118 |
|  | Northwest | Shaanxi | Baoji | 34.37980922 | 107.2031598 |
|  | East | Shandong | Heze | 35.15579967 | 115.6921261 |
|  | East | Shandong | Jinan | 36.73835363 | 117.0920564 |
|  | East | Shandong | Weihai | 37.12465083 | 121.9813021 |
|  | East | Shandong | Weifang | 36.54039692 | 119.0769118 |
|  | East | Shandong | Yantai | 37.24631023 | 120.8073299 |
|  | East | Shandong | Zaozhuang | 34.91917688 | 117.3952413 |
|  | East | Shandong | Zibo | 36.60944072 | 118.057108 |
|  | East | Shanghai | Shanghai | 31.18546101 | 121.4208458 |
|  | North | Shanxi | Jinzhong | 37.3290385 | 112.9639443 |
|  | North | Shanxi | Taiyuan | 37.96112892 | 112.3150466 |
|  | Southwest | Sichuan | Guangyuan | 32.26211873 | 105.7797223 |
|  | Southwest | Sichuan | Panzhihua | 26.80752596 | 101.7312873 |
|  | Southwest | Sichuan | Zigong | 29.28740643 | 104.6827647 |
|  | North | Tianjin | Tianjin | 39.31417415 | 117.3249732 |
|  | Southwest | Tibet | Lhasa | 30.0348938 | 91.09014949 |
|  | Southwest | Tibet | Naqu | 32.88196168 | 89.0347145 |
|  | Southwest | Tibet | Shannan | 28.29975925 | 92.26311112 |
|  | Northwest | Xinjiang | Hami | 43.04140931 | 93.51352336 |
|  | Northwest | Xinjiang | Kezi | 39.70203691 | 75.98811184 |
|  | Southwest | Yunnan | Baoshan | 24.97603196 | 98.97450111 |
|  | Southwest | Yunnan | Yuxi | 24.13864646 | 102.1997581 |
|  | East | Zhejiang | Jinhua | 29.11688048 | 119.954105 |
|  | East | Zhejiang | Shaoxing | 29.7163403 | 120.6319989 |

13- Please provide a diagram that shows the model structure, including how the disease natural history is represented, the process and determinants of disease acquisition, and how the putative intervention could affect the system.

**Response:** Many thanks for the insightful suggestion. We have provided a diagram (S4 Figure) to show the model structure in the revised manuscript:

**S4 Figure. Diagram of the model structure.**

Abbreviations: PM_2.5_ = particulate matter with an aerodynamic diameter less than or equal to 2.5 μm; YLL = years of life lost; GAM=generalized additive model; NAAQS = China’s National Ambient Air Quality Standards; AQG = WHO’s Ambient Air Quality Guidelines; IT = WHO’s interim targets; PGLE=potential gains in life expectancy; AF = attributable fraction.

14- Please provide a complete list of model parameters, including clear and precise descriptions of the meaning of each parameter, together with the values or ranges for each, with justification or the primary source cited, and important caveats about the use of these values noted.

**Response:** Thanks for the advice. We have accordingly included a complete list of model parameters as a supplemental table (S5 Table) in the revision as the following:

**S5 Table. The list of model parameters in this study.**

| **Parameter** | **Range** | **Caveats** |
| --- | --- | --- |
| Years of life lost | 2.4 to 3021.5 | Dependent variable |
| PM_2.5_ (μg/m^3^) | 3.6 to 985.2 |  |
| Long-term trend | 1 to 1461 | Degrees of freedom = 5 per year |
| Day of the week | 1 to 7 |  |
| Public holiday | 0 and 1 |  |
| Temperature (°C) | -28.0 to 36.5 | Degrees of freedom = 6 |
| Relative humidity (%) | 5.0 to 100.0 | Degrees of freedom = 3 |

Abbreviations: PM_2.5_ = particulate matter with an aerodynamic diameter less than or equal to 2.5 μm.

15- Please provide a diagram that shows the model structure.

**Response:** Done as suggested. The diagram is shown below:

**S4 Figure. Diagram of the model structure.**

Abbreviations: PM_2.5_ = particulate matter with an aerodynamic diameter less than or equal to 2.5 μm; YLL = years of life lost; GAM = generalized additive model; NAAQS = China’s National Ambient Air Quality Standards; AQG = WHO’s Ambient Air Quality Guidelines; IT = WHO’s interim targets; PGLE = potential gains in life expectancy; AF = attributable fraction.

16- In the Discussion, please include a paragraph just preceding the Conclusion that discusses implications and next steps for research, clinical practice, and/or public policy.

**Response:** Done as suggested. We have added a new paragraph in the **Discussion** section to discuss the implications and next steps in the future:

“The findings from this study have some important implications for both public health and environment management. We suggest to apply this indictor in future efforts. For example, the PGLE can be applied to estimate the effects of other air pollutants on life expectancy, as well as conducted in different populations. The average life expectancy in China was 76.25 years in 2016. The Chinese government released the Healthy China (HC 2030) blueprint in 2016 as a national strategy. One goal of this plan is to increase the average life expectancy to 79 years by 2030. To achieve that goal, a series of action plans was suggested, such as health education, diet control, sufficient physical exercise [2]. In this respect, our study provided some new evidence that the life expectancy can be prolonged by controlling the concentrations of air pollution, and we suggest this finding should be considered in future policy making.”.

**Comments from the reviewers:**

**Reviewer #1:**

The study examined the associations between ambient PM_2.5_ and years of life lost (YLL) using four-year daily death data (1,226,849 non-accidental deaths) in 72 cities in China. These authors thus estimated the potential gains in life expectancy (PGLE) under the Chinese Ambient Air Quality Standards and WHO's air quality guidelines. They found that ambient PM_2.5_ reduction was significantly associated with decreased YLL and suggested that WHO's AQG should be selected as reference in China.

This paper addresses important public health issues related to the air pollution. Both the research aim and results in the study are important and interesting. However, I have some considerations in methods section:

**Response:** Thanks for the positive and encouraging words. We appreciate your careful reviews and helpful advices. We have extensively amended the manuscript as shown below.

1)	The research divided the 72 cities into the seven regions (Figure 1) and assessed the association between PM_2.5_ and death at seven regional level. Authors state that same region has similar features (meteorological, culture condition etc.). However, the northwest and southwest cover a rather big area which may have big variation for the features and the two big regions only include a few cities data. Considering this issue, I worry about the research results in regional level which may not be good representative for the two regions. Authors may consider using Bayesian spatial geo-statistical model to assess the association as the model can incorporate the spatial dependence and uncertainly?

**Response:** Thank you for the valuable comments. We agree that the northwest and southwest cover a rather big area with great variations for the features but only a few cities were included in this analysis. We have provided the following revisions:

1. Considering the uncertainty and the complex geo-spatial correlation between the cities, we performed a spatial statistical model by adjusting for the longitude and latitude of the cities in the model using a penalized smoothing splines function [3].

In the **Methods** section, we had: “Considering the uncertainty and the complex geo-spatial correlation between the cities, we performed a spatial statistical model by adjusting for the longitude and latitude of the cities in the model using a penalized smoothing splines function [3].”.

In the **Results** section, we added: “The spatial statistical models for the Northwest and Southwest regions, which additionally adjusted for the longitude and latitude of each city, also produced significant effect estimates, with the coefficients of 0.45 (95% CI: 0.31, 0.59) for the Northwest region and 0.63 (95% CI: 0.52, 0.74) for the Southwest region.”.

2. We acknowledged this issue as one limitation in the **Discussion** section: “The smaller number of cities were included in some regions, such as Northwest and Southwest regions, which might have limited the representativeness of these two regions, however, our sensitivity analyses based on a spatial statistical model produced consistent results, suggested that the issue did not affect the result estimate to a great extent.”

2)	These authors used two-stage Bayesian hierarchical statistical models (Generalized linear model with a Gaussian link and Bayesian hierarchical random-effects meta-analysis) to conduct data analysis. I would suggest authors providing more details of modeling such as model formula, goodness-of-fit of model which will help readers to understand the methods.

**Response:** Done as suggested. We have provided model formula in the **Statistical analyses** section and goodness-of-fit in S8 Figure, S9 Figure and S10 Figure accordingly:

In the **Statistical analyses** section, we added: “We controlled for public holidays and day of the week in the form of categorical variables, while long-term and seasonal trends, temperature and relative humidity were adjusted using penalized smoothing splines [4]. A complete list of model parameters was provided as a supplemental table (S5 Table). We selected the model specifications and the degree of freedom (df) for the smoothers according to previous experiences of similar studies [5]. For example, we applied a df of 6 per year for temporal trends to filter out the information at time scales of about two months, a df of 6 for moving average temperature of the current day and previous 3 days (lag_03_) for the potential nonlinear relationship, and 3 df for the same day’s relative humidity. We explored the associations with different lag structures from the current day (lag_0_) up to 3 days before (lag_3_), and we also evaluated the effects of moving averages for the current day and the previous 1, 2 and 3 days (lag_01_, lag_02_, lag_03_). The statistical model can be specified as:

YLL =*β**PM_2.5_ + s(t, df=6/year) + *β*_1_*day of week + *β*_2_*public holidays

+s(temperature, df =6) + s(relative humidity, df=3) +*α*”

In the **Results** section, we had: “Online Supporting Information file S8 Figure, S9 Figure and S10 Figure showed the diagnostic graphs of the model, including the plot of the residuals, the plot of partial autocorrelation function (PACF), and Q-Q plot for six provincial capital cities. These results showed that there were no discernible autocorrelation and patterns in the residuals, suggesting that the models had acceptable goodness of fit.”.

**S8 Figure. The plot of the residuals for six provincial capital cities**

**S9 Figure. The plot of partial autocorrelation function (PACF) for six provincial capital cities**

**S10 Figure. The normal Q-Q plot for provincial capital city.**

3)	Have these authors considered the other factors such as smoking and social economic factors at city level at meta-analysis?

**Response:** Thank you for pointing out this important issue. We have further conducted a meta-regression to examine whether the PM_2.5_-YLL association could be explained by some city-level variables: Gross Domestic Product (GDP), population density, GDP per capita, elevation, precipitation, poverty, education, annual PM_2.5_ concentration, annual CO concentration, annual O_3_ concentration, annual SO_2_ concentration, annual NO_2_ concentration, annual air pressure, annual temperature, and annual relative humidity. However, due to data availability, there are some other city-level factors, such as smoking rate, that cannot be included in this research. The S12 Table in this revision shows the results:

**S12 Table. Change in PM_2.5_–YLL relationship per each IQR increase in city-level variables.**

| City-level variables | IQR | Change in PM_2.5_–YLL relationship (%) | *P*-value |
| --- | --- | --- | --- |
| Gross Domestic Product (in billions, CNY) | 4.98 | 0.10 (-0.22, 0.41) | 0.55 |
| Population density (in millions) | 102.65 | 0.00 (-0.02, 0.03) | 0.81 |
| GDP per capita (in billions, CNY) | 0.025 | 0.02 (-0.05, 0.05) | 0.97 |
| Elevation (m) | 944.70 | -0.32 (-0.97, 0.34) | 0.34 |
| Precipitation (mm) | 66.97 | -0.35 (-0.97, 0.27) | 0.27 |
| Poverty rate (%) | 20.99 | -0.04 (-0.28, 0.2) | 0.74 |
| Education level (by year of school) | 1.71 | -0.14 (-0.46, 0.18) | 0.40 |
| Annual PM_2.5_ concentration (μg/m^3^) | 39.40 | -0.59 (-1.18, 0.00) | <0.05 |
| Annual CO concentration (μg/m^3^) | 129.24 | 0.11 (-0.33, 0.56) | 0.62 |
| Annual O_3_ concentration (μg/m^3^) | 22.58 | -0.34 (-0.73, 0.05) | 0.09 |
| Annual SO_2_ concentration (μg/m^3^) | 25.61 | -0.01 (-0.31, 0.29) | 0.94 |
| Annual NO_2_ concentration (μg/m^3^) | 16.12 | -0.01 (-0.46, 0.43) | 0.95 |
| Annual air pressure (kPa) | 69.42 | -0.11 (-0.66, 0.45) | 0.70 |
| Annual temperature (℃) | 6.33 | 0.27 (-0.1, 0.64) | 0.15 |
| Annual relative humidity (%) | 18.06 | -0.11 (-0.68, 0.47) | 0.72 |

Abbreviations: PM_2.5_ = particulate matter with an aerodynamic diameter less than or equal to 2.5 μm; YLL = years of life lost; IQR = the difference between the third quartile and the first quartile; CNY = Chinese Yuan; CO = carbon monoxide; O_3_ = ozone; SO_2_ = sulfur dioxide; NO_2_ = nitrogen dioxide.

4)	Line 138 GAM or GLM?

**Response:** Thanks for the careful review. We clarified that it was the generalized additive model (GAM) being used in our analyses as we included penalized smoothing spline variables in the model.

**Reviewer #2:**

This study estimates the potential benefits on life expectancy by the daily ambient PM_2.5_ concentration meets the Chinese and WHO air quality standard respectively in both national and regional levels of China. The result provides a useful information for policy making on quantifying the beneficial effects of air pollution reduction. This manuscript is suitable to be published in PLOS Medicine after the following revision is conducted.

**Response:** Thank you for the positive comments and valuable revision suggestions. We have addressed each of the specific comments as below.

General comments:

Dataset includes concentrations of PM_2.5_, SO_2_, and NO_2_ and meteorological parameters such as temperature and relative humidity. However, very few discussions have been made using those air pollutants and meteorological parameters. Only a simple correlation results are shown in Table S1, so the meaning to use the above dataset in this paper is not clear. If this paper includes those data more deeply analysis and discussion is needed.

**Response:** Thank you for raising this concern. The meteorological factors have been adjusted in the model using a smoothing function. As suggested, in this revision we have conducted two-pollutant models to control for the potential confounding effects of O_3_, SO_2_, and NO_2_. We have further explained them in the **Results** and **Discussion** sections:

We gave more descriptions on the correlation results in the **Results** section: “The correlation analyses showed low to moderate correlation coefficients between air pollutants and weather variables. For example, PM_2.5_ had moderate positive correlations with NO_2_ (correlation coefficient=0.50), had relatively lower correlations with SO_2_ and O_3_ (correlation coefficients of 0.26 and 0.29, respectively), and had a negative correlation with mean temperature and relative humidity (correlation coefficients of -0.15 and -0.02, respectively) (S6 Table).”.

The results of two-pollutant models were presented in the **Results** section: “In the two-pollutant models, the significant associations between PM_2.5_ and YLL generally remained (S7 Table). For instance, at the national level, each 10 μg/m^3^ increase in lag_02_ PM_2.5_ concentration was associated with an increment of 0.41 (95% CI: 0.27, 0.55), 0.32 (95% CI: 0.19, 0.45) or 0.41 (95% CI: 0.27, 0.55) in YYL after controlling for SO_2_, NO_2_ and O_3_, respectively.”.

In the **Discussion** section, we had: “The observed associations between PM_2.5_ and YLL were generally robust in the sensitivity analyses. In particular, the associations remained consistent in the two-pollutant models with adjustment for other air pollutants, indicating that the associations were not confounded by these air pollutants. However, we observed a relatively smaller estimate when adjusting for NO_2_, which could be partly explained by the moderate positive correlation between PM_2.5_ and NO_2_ (r = 0.50). It was also possible that PM_2.5_ and NO_2_ shared similar emission sources and biological pathways in their health effects [6, 7].”.

The 72 cities are divided into 7 regions in this study. However, there is almost no discussion on the comparison and interpretation about the different results of PGLE, Avoidable YLL and AF among those regions.

**Response:** Thanks for your careful review. We have added more information in the **Results** and **Discussion** section to discuss the different results in these regions in this study:

In the **Results** section, we had: “The region-specific results showed that the associations varied by regions. For example, Northwest region was found to have the highest association (β=0.94, 95% CI: 0.21, 1.68), while North region had the lowest association with a regression coefficient of 0.12 (95% CI: 0.03, 0.22).” **and** “The spatial statistical models for the Northwest and Southwest regions, which additionally adjusted for the longitude and latitude of each city, also produced significant effect estimates, with the coefficients of 0.45 (95% CI: 0.31, 0.59) for the Northwest region and 0.63 (95% CI: 0.52, 0.74) for the Southwest region. While the one-stage mix-effect model also yielded a significant effect [the overall regression coefficient was 0.18 (95% CI: 0.09, 0.27)], though the estimate was relatively smaller (S11 Table).” **and** “In general, we observed higher effect estimates when adopting stricter air quality standards. Heterogeneity of the estimates was observed across the seven regions. For example, when adopting the WHO’s AQG, the East region was found to have the largest avoidable YLL (46572.93 YLLs), while the North region had the lowest avoidable YLL (22617.94 YLLs).” **and** “Among the seven regions, the largest value of 0.28 (95% CI: 0.06, 0.49) was observed in the Northwest region and the minimum value of 0.08% (95% CI: 0.02, 0.15) was found in the North region by using WHO’s AQG as the reference.”.

In the **Discussion** section, we added: “We observed some evidence for spatial heterogeneity in the association between PM_2.5_ and YLL across different regions of China. This finding was in line with previous studies [8]. Generally, we found relatively weaker associations in the North, East, and South regions, whereas the associations were stronger in Northwest and Southwest regions. The underlying reasons remained unclear. One possible underlying reason might be the differences in emission sources and chemical constituents of ambient PM_2.5_ among the different regions. The PM_2.5_ in the Northwest and Southwest regions may be more hazardous than that in other regions, much of the ambient fine particles were related to biomass combustion, which was more toxic than other sources [9]. Our meta-regression analysis showed that the areas with higher annual concentrations of PM_2.5_ tended to have a lower PM_2.5_–YLL association, indicating a better adaptation to the local environmental conditions in the areas with higher levels of air pollution. it was possible that people living in highly polluted areas have a higher self-protection awareness, which could lead to a better protection activity, such as wearing masks, reducing outdoor activities, and use of air purifiers [8].”.

The English of this manuscript needs to be revised by native speaker.

**Response:** Thanks for the comments. We have invited a native English speaker with expertise in environmental epidemiology to polish the language. We hope all the language problems have been amended.

Specific comments:

Line 50: "air pollution" is no necessary shown as Keywords.

**Response:** Removed as suggested.

Line 64-65: Data sources needs to be cited for annual PM_2.5_ concentration.

**Response:** Done. We have added the reference for the data sources of the mentioned annual PM_2.5_ concentration.

Line 103: What does the "randomly select and extract" mean?

**Response:** Thank you. We have provided more detailed of the selection of the cities:

These cities were selected based on the following process: (1) they were randomly selected using a multistage stratification approach that took the sociodemographic characteristics of the Chinese population into consideration; (2) the daily morality counts in these cities were temporally stable without large fluctuations, and no change in the administrative divisions occurred during the study period; and (3) their air pollution and meteorological records were accessible during the study period.

Line 105: How to check the data set needs to be described more detailed.

**Response:** Thanks for the suggestion. We added more details about the data collection process: “Practitioners in the health facilities were responsible for checking of the accuracy, completeness, and data quality of the death’s data, and they then reported that information to the Death Surveillance Points System. Staff in the district-level CDC reviewed all new information to ensure the data quality (i.e., to check that the ICD codes were maintained and to exclude the duplicate records and redundant information) in the system within 7 days, as well as returned the unclear or uncertain records back to the reporting health facilities. Then, practitioners in those health facilities asked the physicians to correct and confirm the data. Staff in district-level CDC also collected non-accidental death information from the security department (the other government department collecting the death information for the purpose of residence) every month. Then, the staff of the provincial- or regional-level CDC would conduct a second round of checking and reviewing. Finally, data were sent to the national-level CDC to undergo a further round of review, which included the duplication, logic, data analysis, and investigation of misreported data.”.

Line 195-196: How to interpret this correlation results? What does the slightly positive or negative correlation mean?

**Response:** Thanks for raising this issue. In this revision, we have provided more specific interpretations of this correlation results in the **Results** and **Discussion** section:

In the **Results** section, we added: “The correlation analyses showed low to moderate correlation coefficients between air pollutants and weather variables. For example, PM_2.5_ had moderate positive correlations with NO_2_ (correlation coefficient=0.50), had relatively lower correlations with SO_2_ and O_3_ (correlation coefficients of 0.26 and 0.29, respectively), and had a negative correlation with mean temperature and relative humidity (correlation coefficients of -0.15 and -0.02, respectively) (S6 Table).”

In the **Discussion** section, we had: “The observed associations between PM_2.5_ and YLL were generally robust in the sensitivity analyses. In particular, the associations remained consistent in the two-pollutant models with adjustment for other air pollutants, indicating that the associations were not confounded by these air pollutants. However, we observed a relatively smaller estimate when adjusting for NO_2_, which could be partly explained by the moderate positive correlation between PM_2.5_ and NO_2_ (r = 0.50). It was also possible that PM_2.5_ and NO_2_ shared similar emission sources and biological pathways in their health effects [6, 7].”.

Table 1: The difference of those variables among the 7 regions needs to be interpreted and discussed in main text. I think population is also important factor for health risk assessment. Why there is no data and discussion on population in different regions?

**Response:** Thanks. We further revised the description of Table 1: “Table 1 summarizes the number of cities, daily mean air pollutant concentrations, meteorological conditions, daily mean mortality and YLL of these regions. The daily mean concentrations of PM_2.5_, SO_2_, NO_2_ and O_3_ ranged from 49.29 to 95.90, 27.15 to 53.94, 26.80 to 48.99, and 66.75 to 100.69, respectively. The mean temperature ranged from 7.35 °C to 21.84 °C, and relative humidity ranged from 49.31% to 77.79%. Moreover, the daily mean YLLs were 87.16, 123.44, 146.09, 145.04, 158.06, 121.37 and 179.78 years in the seven regions of Northwest, North, Northeast, Central, East, Southwest and South regions, respectively.”.

We agree with the reviewer that population is another important factor, as such, we included the population density in the meta-regression model to examine whether the observed differential PM_2.5_-YLL associations across the cities could be explained by some city-level variables including population density. However, among various potential explanatory variables, we only found that annual mean PM_2.5_ concentration was significant, while population density and other variables could not explain the differential associations across the cities.

In the **Methods** section, we added: “In addition, we performed a meta-regression to evaluate whether the observed PM_2.5_–YLL relationship could be explained by some city-level variables: Gross Domestic Product (GDP), population density, GDP per capita, elevation, precipitation, poverty, education, annual PM_2.5_ concentration, annual CO concentration, annual O_3_ concentration, annual SO_2_ concentration, annual NO_2_ concentration, air pressure, annual temperature, and annual relative humidity.”

In the **Results** section, we added: “In addition, we evaluated whether the observed PM_2.5_–YLL relationship could be explained by some city level factors (S12 Table). The analysis showed that the associations between PM_2.5_ and YLL were relatively higher in cities with lower annual mean concentrations of PM_2.5_. Each IQR (39.40 μg/m^3^) increase in annual concentrations of PM_2.5_ was associated with a 0.59 decrease in the regression coefficient. While we did not find any significant effect modification by other variables.”.

Line 211: After control NO_2_ the increment of YYL is smaller than control SO_2_ and O_3_. The reason needs to be interpreted.

**Response:** Thanks for the insightful comment. We have added one more paragraph in the **Discussion** section to interpret the reason: “The observed associations between PM_2.5_ and YLL were generally robust in the sensitivity analyses. In particular, the associations remained consistent in the two-pollutant models with adjustment for other air pollutants, indicating that the associations were not confounded by these air pollutants. However, we observed a relatively smaller estimate when adjusting for NO_2_, which could be partly explained by the moderate positive correlation between PM_2.5_ and NO_2_ (r = 0.50). It was also possible that PM_2.5_ and NO_2_ shared similar emission sources and biological pathways in their health effects [6, 7].”.

Line 229-231: The description of "reducing reference standard" is not clear.

**Response:** Thank you for your careful review. This sentence has been revised to: “In general, we observed higher effect estimates when adopting stricter air quality standards. Heterogeneity of the estimates was observed across the seven regions. For example, when adopting the WHO’s AQG, the East region was found to have the largest avoidable YLL (46572.93 YLLs), while the North region had the lowest avoidable YLL (22617.94 YLLs).”.

Line 246-250: The reason and indication from this different result of YLL in 7 regions needs to be interpreted.

**Response:** Thank you. We have added more interpretations in the **Results** and **Discussion** section to discuss the underlying potential reasons and indications from the different results of YLL in the 7 regions:

In the **Results** section, we had: “The region-specific results showed that the associations varied by regions. For example, Northwest region was found to have the highest association (β=0.94, 95% CI: 0.21, 1.68), while North region had the lowest association with a regression coefficient of 0.12 (95% CI: 0.03, 0.22).” **and** “The spatial statistical models for the Northwest and Southwest regions, which additionally adjusted for the longitude and latitude of each city, also produced significant effect estimates, with the coefficients of 0.45 (95% CI: 0.31, 0.59) for the Northwest region and 0.63 (95% CI: 0.52, 0.74) for the Southwest region. While the one-stage mix-effect model also yielded a significant effect [the overall regression coefficient was 0.18 (95% CI: 0.09, 0.27)], though the estimate was relatively smaller (S11 Table).” **and** “In general, we observed higher effect estimates when adopting stricter air quality standards. Heterogeneity of the estimates was observed across the seven regions. For example, when adopting the WHO’s AQG, the East region was found to have the largest avoidable YLL (46572.93 YLLs), while the North region had the lowest avoidable YLL (22617.94 YLLs).” **and** “Among the seven regions, the largest value of 0.28 (95% CI: 0.06, 0.49) was observed in the Northwest region and the minimum value of 0.08% (95% CI: 0.02, 0.15) was found in the North region by using WHO’s AQG as the reference.”.

In the **Discussion** section, we added: “We observed some evidence for spatial heterogeneity in the association between PM_2.5_ and YLL across different regions of China. This finding was in line with previous studies [8]. Generally, we found relatively weaker associations in the North, East, and South regions, whereas the associations were stronger in Northwest and Southwest regions. The underlying reasons remained unclear. One possible underlying reason might be the differences in emission sources and chemical constituents of ambient PM_2.5_ among the different regions. The PM_2.5_ in the Northwest and Southwest regions may be more hazardous than that in other regions, much of the ambient fine particles were related to biomass combustion, which was more toxic than other sources [9]. Our meta-regression analysis showed that the areas with higher annual concentrations of PM_2.5_ tended to have a lower PM_2.5_–YLL association, indicating a better adaptation to the local environmental conditions in the areas with higher levels of air pollution. it was possible that people living in highly polluted areas have a higher self-protection awareness, which could lead to a better protection activity, such as wearing masks, reducing outdoor activities, and use of air purifiers [8].”.

**Reviewer #3:**

Thank you for the opportunity to review this paper. Lin and colleagues conducted a nationwide study in China evaluating the association between daily PM_2.5_, years of life lost and further modelled potential gains in life due to achieving targeted national and WHO standards of PM_2.5_ targets. They have found across China, although with variation in levels of potential gains in life expectancy across regions, that overall significant gains in average life expectancy could be made by further reducing concentration levels. The 2-stage modelling approach is commonly used in ecological studies with the first stage estimating association using a generalised linear model (link function in this case was Gaussian) between PM_2.5_ exposure and outcome of YLL. The second stage utilised a random-effects meta-analysis to tool cities and regions to obtain a national average. The authors also explored 3 day lag structure and moving averages, confounding by temperature and humidity. The study is well-conducted and presented. The only major issue I would have liked to have seen more thought given to in the analysis is incorporation other population level covariates which may affect this association - education, urbanicity, smoking, per capita income, unemployment come to mind.

**Response:** Thanks for the encouraging comment on our manuscript and we appreciate the careful reviews and helpful advices very much.

As suggested, we conduct a meta-regression model to whether some population-level variables could affect the observed PM_2.5_-YLL associations.

In the **Methods** section, we added: “In addition, we performed a meta-regression to evaluate whether the observed PM_2.5_–YLL relationship could be explained by some city-level variables: Gross Domestic Product (GDP), population density, GDP per capita, elevation, precipitation, poverty, education, annual PM_2.5_ concentration, annual CO concentration, annual O_3_ concentration, annual SO_2_ concentration, annual NO_2_ concentration, air pressure, annual temperature, and annual relative humidity.”.

In the **Results** section, we had: “In addition, we evaluated whether the observed PM_2.5_–YLL relationship could be explained by some city level factors (S12 Table). The analysis showed that the associations between PM_2.5_ and YLL were relatively higher in cities with lower annual mean concentrations of PM_2.5_. Each IQR (39.40 μg/m^3^) increase in annual concentrations of PM_2.5_ was associated with a 0.59 decrease in the regression coefficient. While we did not find any significant effect modification by other variables.”.

In the **Discussion** section, we added: “Our meta-regression analysis showed that the areas with higher annual concentrations of PM_2.5_ tended to have a lower PM_2.5_–YLL association, indicating a better adaptation to the local environmental conditions in the areas with higher levels of air pollution. It was possible that people living in highly polluted areas have a higher self-protection awareness, which could lead to a better protection activity, such as wearing masks, reducing outdoor activities, and use of air purifiers [8].”.

1) Abstract methods do not mention the method on using adopting a two-stage model to combine city-specific results to estimate the nation-wide mean potential years of life expectancy gained.

**Response:** Done. We have revised the relevant text in **Abstract Methods** to: “We applied a two-stage approach for the analysis. At the first stage, we used a generalized additive model with the Gaussian link to examine the city-specific short-term association between daily PM_2.5_ and years of life lost (YLL); at the second stage, a random-effects meta-analysis was used to generate the regional and national estimations. We further estimated the potential gains in life expectancy by assuming that ambient PM_2.5_ has met the Chinese National Ambient Air Quality Standard (75 μg/m^3^) or the Ambient Air Quality Guideline of the World Health Organization (25 μg/m^3^).”.

2) Abstract methods - would be helpful to indicate what is the recommended level of ambient PM_2.5_ by the Chinese National air quality threshold and the WHO threshold.

**Response:** Done as suggested. We added the recommended levels in the **Abstract and Methods sections**: “We further estimated the potential gains in life expectancy by assuming that ambient PM_2.5_ has met the Chinese National Ambient Air Quality Standard (75 μg/m^3^) or the Ambient Air Quality Guideline of the World Health Organization (25 μg/m^3^).”.

3) Abstract conclusions - it's unclear what the authors mean by "PM_2.5_ might be a non-ignored risk factor" as national and international policy is currently targeting reduction in PM_2.5_ to reduce premature deaths.

**Response:** We are sorry for this confusion. That sentence has been revised to: “This study indicates that significantly longer life expectancy could be achieved by a reduction in the ambient PM_2.5_ concentrations.”.

4) Intro lines 77-81. It would be helpful for the reader to clearly delineate between short-term exposure and long-term exposure PM_2.5_ and what the suggested mechanism is for reduction of life expectancy and how they may differ from one another (i.e. some literature suggests long-term exposure has bigger effects).

**Response:** Thanks for the insightful comment. We have accordingly revised the relevant text to delineate the difference between short-term and long-term exposure to PM_2.5_: “Previous studies have examined the association between short-term and long-term exposures to ambient air pollution and mortality or YLL. The short-term studies, usually based on the daily time series data, evaluated the acute health effects of air pollution [10], while the long-term studies estimated health effects of chronic and cumulative air pollution exposures (usually average concentration of several years as the exposure indicator) [11]. The long-term health effect studies generally reported relative larger effects than that in short-term analyses [12]. The exact biological mechanisms for the health effects of PM_2.5_ exposure remained unclear, previous studies suggested that oxidative stress, systemic inflammation, direct vascular endothelial impairment, and alterations in arterial tone might have played important roles [13-15].”.

5) Intro lines 89-92. YLL is still very commonly used and has been extended by the WHO recommended DALY measure combining mortality and morbidity. Influence by age size of the population can be solved by age-direct standardisation approaches.

see https://academic.oup.com/ije/article/48/4/1367/5281229

To me, the main advantage of PGLE isn't the influence of age structure and size as standardisation techniques allow for comparisons across difference areas, but rather the fact that YLL is extremely sensitive to competing risks (that is the exposure of interest contributes to several causes of death). This has not been mentioned in the text.

**Response:** We appreciate the careful reviews and have benefited a lot from these valuable suggestions. In this revision, we have discussed this main advantage as suggested.

In the **Introduction** section, we had: “Through directly quantifying the health benefits by attaining the air quality standards, PGLE is more relevant to air pollution controlling and formulation of air quality standards. Another advantage of PGLE was that it can be easily compared across different areas, while excess deaths and YLL were somewhat influenced by the age structure and size of the study population [16]. Although this limitation can be solved by several standardization techniques, the YLL was subject to one important issue of its sensitivity to competing risks of death [16, 17]”.

6) Methods Line 103 - What is the rationale here for random selection? I would have thought that it would be more advantageous to consider stratified sampling strategy to have an equal coverage across regions. Was this a pragmatic decision due to the number of cities which needed to be reduced or had a priori rationale.

**Response:** Thanks for raising this comment. We have provided a detailed description on the selection of the cities in the revised manuscript, which included a stratified sampling strategy to ensure adequate representation of the national data:

“These cities were selected based on the following process: (1) they were randomly selected using a multistage stratification approach that took the sociodemographic characteristics of the Chinese population into consideration; (2) the daily morality counts in these cities were temporally stable without large fluctuations, and no change in the administrative divisions occurred during the study period; and (3) their air pollution and meteorological records were accessible during the study period.”.

7) Methods line 167 on two-stage Bayesian hierarchical model. This is an appropriate approach, where in stage 1 GLM with Gaussian link to estimate city specific association between PM_2.5_ and YLL, then using second stage to include a random-effect terms to pool cities to create regional and national estimates.

My question related to whether authors also consider a fully specified one-stage models (random study intercept or fixed study-specific intercept; random exposure effect; and fixed study-specific effects for covariate), investigating interaction - and if using a 1-stage this would have impacted the results instead of a 2-stage model.

**Response:** Special thanks for your good comments. We have conducted the one-stage models with random effect, and the results are showed in S11 Table.

In the **Methods**, we added: “Furthermore, we also used a mixed-effect generalized additive model as a one-stage approach to examine the regional and national estimates, where we included the variable of city as a random term.”.

In the **Results**, we had: “While the one-stage mix-effect model also yielded a significant effect [the overall regression coefficient was 0.18 (95% CI: 0.09, 0.27)], though the estimate was relatively smaller (S11 Table).”.

The results were showed as below:

**S11 Table. Sensitivity analyses for the absolute change in years of life lost associated with each 10 μg/m^3^ increase in PM_2.5_ in different models.**

| **Regions** | **Model 1** | **Model 2** |
| --- | --- | --- |
| Northwest | 0.94 (0.21, 1.68) | 0.76 (0.35, 1.18) |
| North | 0.12 (0.03, 0.22) | 0.08 (-0.04, 0.19) |
| Northeast | 0.43 (0.05, 0.81) | 0.43 (0.22, 0.64) |
| Central | 0.61 (0.05, 1.17) | 0.23 (-0.12, 0.58) |
| East | 0.37 (0.13, 0.61) | 0.33 (0.15, 0.52) |
| Southwest | 0.85 (0.60, 1.09) | 0.68 (0.44, 0.93) |
| South | 0.58 (0.25, 0.92) | -0.07 (-0.47, 0.33) |
| National | 0.43 (0.29, 0.57) | 0.18 (0.09, 0.27) |

Model 1: Two-stage model which generating the regional and national estimates by meta-analysis;

Model 2: Mix-effects regression model adjusting for the variable of city with a random term.

8) Figure 2 - northwest region (n=8) missing reference line for 0.

**Response:** We are sorry for this mistake. The reference line for 0 has been added in Figure 2.

**Fig 2. Associations between ambient PM_2.5_ and years of life lost in seven regions of mainland China, 2013–2016.**

9) Figure 3 needs better labelling y axis label missing and x-axis labels shared common labels between vertical panels are not intuitive. Also some bars are missing what I assume are the 95% CIs

**Response:** Corrected as suggested. The revised figure is showed below:

**Fig 3. The estimated potential gains in life expectancy by attaining the WHO’s air quality guidelines and their interim targets in seven regions of mainland China, 2013–2016** (Based on the effects of moving averaged concentration of lag 0 to lag 2 (lag_02_) of daily PM_2.5_)

**References:**

1.	Liu S, Wu X, Lopez AD, Wang L, Cai Y, Page A, et al. An integrated national mortality surveillance system for death registration and mortality surveillance, China. Bull World Health Organ. 2016;94(1):46-57. Epub 10/28. doi: 10.2471/BLT.15.153148. PubMed PMID: 26769996.

2.	Tan X, Liu X, Shao H. Healthy China 2030: A Vision for Health Care. Value in health regional issues. 2017;12:112-4. doi: 10.1016/j.vhri.2017.04.001. PubMed PMID: 28648308.

3.	Guo Y, Zeng H, Zheng R, Li S, Barnett AG, Zhang S, et al. The association between lung cancer incidence and ambient air pollution in China: A spatiotemporal analysis. Environmental research. 2016;144:60-5.

4.	Lin H, Tao J, Du Y, Liu T, Qian Z, Tian L, et al. Particle size and chemical constituents of ambient particulate pollution associated with cardiovascular mortality in Guangzhou, China. Environ Pollut. 2016;208(Pt B):758-66. doi: 10.1016/j.envpol.2015.10.056. PubMed PMID: 26561449.

5.	Tian L, Qiu H, Pun VC, Lin H, Ge E, Chan JC, et al. Ambient Carbon Monoxide Associated with Reduced Risk of Hospital Admissions for Respiratory Tract Infections. Am J Respir Crit Care Med. 2013;188(10):1240-5. Epub 2013/08/16. doi: 10.1164/rccm.201304-0676OC. PubMed PMID: 23944864.

6.	Rohde RA, Muller RA. Air Pollution in China: Mapping of Concentrations and Sources. PLoS One. 2015;10(8):e0135749. doi: 10.1371/journal.pone.0135749.

7.	Liang Z, Yang Y, Qian Z, Ruan Z, Chang J, Vaughn MG, et al. Ambient PM_2.5_ and birth outcomes: Estimating the association and attributable risk using a birth cohort study in nine Chinese cities. Environ Int. 2019;126:329-35. doi: <https://doi.org/10.1016/j.envint.2019.02.017>.

8.	Chen R, Yin P, Meng X, Liu C, Wang L, Xu X, et al. Fine Particulate Air Pollution and Daily Mortality. A Nationwide Analysis in 272 Chinese Cities. Am J Respir Crit Care Med. 2017;196(1):73-81. doi: 10.1164/rccm.201609-1862OC.

9.	Krall Jenna R, Mulholland James A, Russell Armistead G, Balachandran S, Winquist A, Tolbert Paige E, et al. Associations between Source-Specific Fine Particulate Matter and Emergency Department Visits for Respiratory Disease in Four U.S. Cities. Environ Health Perspect. 2017;125(1):97-103. doi: 10.1289/EHP271.

10.	Zhang L, Yang Y, Li Y, Qian Z, Xiao W, Wang X, et al. Short-term and long-term effects of PM_2.5_ on acute nasopharyngitis in 10 communities of Guangdong, China. Sci Total Environ. 2019;688:136-42. doi: <https://doi.org/10.1016/j.scitotenv.2019.05.470>.

11.	Cai Y, Zhang B, Ke W, Feng B, Lin H, Xiao J, et al. Associations of Short-Term and Long-Term Exposure to Ambient Air Pollutants With Hypertension. Hypertension. 2016;68(1):62-70. doi: 10.1161/HYPERTENSIONAHA.116.07218.

12.	Liang F, Xiao Q, Gu D, Xu M, Tian L, Guo Q, et al. Satellite-based short- and long-term exposure to PM_2.5_ and adult mortality in urban Beijing, China. Environ Pollut. 2018;242:492-9. doi: <https://doi.org/10.1016/j.envpol.2018.06.097>.

13.	Brook Robert D, Brook Jeffrey R, Urch B, Vincent R, Rajagopalan S, Silverman F. Inhalation of Fine Particulate Air Pollution and Ozone Causes Acute Arterial Vasoconstriction in Healthy Adults. Circulation. 2002;105(13):1534-6. doi: 10.1161/01.CIR.0000013838.94747.64.

14.	Zhong J, Urch B, Speck M, Coull Brent A, Koutrakis P, Thorne Peter S, et al. Endotoxin and β-1,3-d-Glucan in Concentrated Ambient Particles Induce Rapid Increase in Blood Pressure in Controlled Human Exposures. Hypertension. 2015;66(3):509-16. doi: 10.1161/HYPERTENSIONAHA.115.05342.

15.	Mills NL, Miller MR, Lucking AJ, Beveridge J, Flint L, Boere AJF, et al. Combustion-derived nanoparticulate induces the adverse vascular effects of diesel exhaust inhalation. Eur Heart J. 2011;32(21):2660-71. doi: 10.1093/eurheartj/ehr195.

16.	Lai D, Hardy RJ. Potential gains in life expectancy or years of potential life lost: impact of competing risks of death. Int J Epidemiol. 1999;28(5):894-8. doi: 10.1093/ije/28.5.894.

17.	Martinez R, Soliz P, Caixeta R, Ordunez P. Reflection on modern methods: years of life lost due to premature mortality—a versatile and comprehensive measure for monitoring non-communicable disease mortality. Int J Epidemiol. 2019;48(4):1367-76. doi: 10.1093/ije/dyy254.
