# Supplementary material for: Potential gains in life expectancy by attaining daily ambient fine particulate matter pollution standards in mainland China: A modeling study based on nationwide data
Source: PLoS Med. 2020 Jan 17;17(1):e1003027. doi: 10.1371/journal.pmed.1003027 (PMC6968855; doi:10.1371/journal.pmed.1003027)
Supplement: Attachment — Submitted filename: 2 Response_20191114_R2.docx [file pmed.1003027.s015.docx]

**Response to the comments from the Editor and Reviewers**

Dear Editor and Reviewers,

Thank you very much for the valuable comments concerning our manuscript. We appreciate the opportunity to further revise the manuscript. We have addressed each of the comments carefully and have made corresponding revisions accordingly. The specific point-to-point responses are shown below.

**Requests from the editors:**

1- Thank you for including an Author Summary. The last point of the What did the researchers do and find? Section (“By adopting the WHO’s guideline as a reference, we estimated that about 1.00% of the years of life lost could be avoided in the study area.”) could use a bit more context or explanation. How is this point different from the point that precedes it? Over what timeframe is this estimate? Perhaps the point can be removed.

**Response:** Thanks. As suggested, we removed the last point, and revised the third point into: “During the study period from January 18, 2013 to December 31, 2016, we estimated that 168065.18 (about 1.00% of the total) years of life lost can be avoided by achieving the WHO’s guideline on daily PM_2.5_ concentrations (25 μg/m^3^) in the study area, which corresponded to 0.14 years of gains in life expectancy for each death.”.

2- The Abstract Methods and Findings should also include the results for the Chinese NAAQS projection, quoted around line 376.

**Response:** Done as suggested. The relevant text has been revised to “We estimated that 168065.18 (95% CI: 114144.91, 221985.45) and 68684.95 (95% CI: 46648.79, 90721.11) years of life lost can be avoided by achieving the WHO’s AQG and Chinese NAAQS in the study area, which corresponded to 0.14 (95% CI: 0.09, 0.18) and 0.06 (95% CI: 0.04, 0.07) years of gain in life expectancy for each death in these cities.” and “Furthermore_,_ using WHO’s AQG or Chinese NAAQS as the references, we estimated that 1.00% (95% CI: 0.68%, 1.32%) or 0.41% (95% CI: 0.28%, 0.54%) of YLLs could be attributable to the PM_2.5_ exposure at the national level.”.

3- Line 480 do you mean “neglected” instead of “non-ignored”?

**Response:** Thanks. As suggested, that sentence has been revised to “In conclusion, this study indicates that ambient PM_2.5_ might be a risk factor of YLL that should not be neglected, and significantly longer life expectancy could be achieved by a reduction in the pollution level.”.

4- Please make sure all references use the "Vancouver" style for reference formatting, and see our website for other reference guidelines https://journals.plos.org/plosmedicine/s/submission-guidelines#loc-references

**Response:** Done. We have re-formatted all references according to the reference guidelines.

**Comments from the reviewers:**

**Reviewer #1:** I think the manuscript has been improved. However, I have little consideration for the spatial model. These authors stated "A spatial statistical model by adjusting for the longitude and latitude of the cities in the model using a penalized smoothing splines function." I would suggest mapping these residuals from the model and check if these residuals are independence or appear spatial auto-correlation. Some discussion for why need to control the spatial auto-correlation in spatial model is needed.

**Response:** Thanks and done as suggested. We showed the plot of the residuals of the model as supporting information, which suggested that these residuals are independence and there were no obviously discernible auto-correlation and patterns.

In the **Results**, we had: “The plot of residuals at the national level suggested that these residuals are independence and there were no obviously discernible auto-correlation and patterns (S11 Figure).”.

Moreover, as suggested, we added the following discussion in the manuscript: “Furthermore, spatial auto-correlation might be one issue in this analysis, however, this concern should be minimal as the cities were sparsely distributed in different areas, and our spatial model controlling for longitude and latitude of the cities yielded a consistent result.”.

**Reviewer #2:** I am appreciated to have a chance to review this paper again.

The authors have answered my comments sufficiently and revised the manuscript accordingly.

I have no more comment.

There are many comments about the models from other reviewers who are experts on this field. I am not sure whether the reply and revision is sufficient or not.

If the answer and revision about the method can be acceptable, this paper is suitable to be publish.

**Response:** Thanks for the positive and encouraging comments.

**Reviewer #3:** Thank you for addressing issues raised by in the reviews and incorporating additional analyses using the suggested methods. With these additional analyses, it's clear now that the primary results are robust (after numerous sensitivity/stress tests). There were some differences (i.e. use of one-stage model resulted in smaller effect sizes but the result retained significance), but the authors were able to detail and interpret these findings appropriately.

For me, the key strength now of the analyses is the separate meta-regression analyses exploring population level confounders. The interesting highlight of the confounders was the annual concentration being higher in areas had smaller effects between PM_2.5_ and life years. The authors suggest this could be self-awareness in areas with higher annual levels of pollution. I agree this could play a role but the final suggestion I think would be worthwhile to check is if there an interaction between annual PM_2.5_ and GDP with more industrialised areas being also wealthier. If so, this could partially explain as well whether there is some protection afforded by being located in an area with better health care access and care. This is a minor point though, so for that reason I recommend proceeding to acceptance of the very interesting and well-conducted analyses.

**Response:** Many thanks for the insightful and positive comments.

As suggested, we conducted an additional analysis which included the interactive term of PM_2.5_ and GDP in the meta-regression model to check the potential interaction effect of the two variables, and no significant interaction was found (p=0.89).

At the same time, we added more interpretations in the **Discussion** section: “Moreover, considering that the cities with a higher PM_2.5_ concentration may also be wealthier and have better health care access, we cannot rule out the possibility that there may be some protective effect of economic development level. We therefore included the interactive term of PM_2.5_ and GDP in the meta-regression model, and did not find a significant interactive effect. Additionally, in the light of previous studies which reported varying effects of PM_2.5_ constituents on human health, we suspected that the differences in chemical component of PM_2.5_ in different areas may be a potential explanation”.
